# Solutions for Lithium Battery Materials Data Issues in Machine Learning: Overview and Future Outlook

**DOI:** 10.1002/advs.202410065

**Published:** 2024-11-18

**Authors:** Pengcheng Xue, Rui Qiu, Chuchuan Peng, Zehang Peng, Kui Ding, Rui Long, Liang Ma, Qifeng Zheng

**Affiliations:** ^1^ School of Chemistry Guangzhou Key Laboratory of Materials for Energy Conversion and Storage South China Normal University Guangzhou 510006 China; ^2^ School of Energy and Power Engineering Huazhong University of Science and Technology Wuhan 430074 P. R. China

**Keywords:** data processing strategies, domain knowledge, lithium battery materials, machine learning

## Abstract

The application of machine learning (ML) techniques in the lithium battery field is relatively new and holds great potential for discovering new materials, optimizing electrochemical processes, and predicting battery life. However, the accuracy of ML predictions is strongly dependent on the underlying data, while the data of lithium battery materials faces many challenges, such as the multi‐sources, heterogeneity, high‐dimensionality, and small‐sample size. Through the systematic review of the existing literatures, several effective strategies are proposed for data processing as follows: classification and extraction, screening and exploration, dimensionality reduction and generation, modeling and evaluation, and incorporation of domain knowledge, with the aim to enhance the data quality, model reliability, and interpretability. Furthermore, other possible strategies for addressing data quality such as database management techniques and data analysis methodologies are also emphasized. At last, an outlook of ML development for data processing methods is presented. These methodologies are not only applicable to the data of lithium battery materials, but also endow important reference significance to electrocatalysis, electrochemical corrosion, high‐entropy alloys, and other fields with similar data challenges.

## Graphical abstract

The lithium battery materials suffer from serious data challenges of multi‐sources, heterogeneity, high‐dimensionality, and small‐sample size for machine learning. Through a systematic review of the existing literatures, effective data processing strategies, including classification and extraction, screening and exploration, dimensionality reduction and generation, modeling and evaluation, and incorporation of domain knowledge, are proposed to accelerate the development of machine learning in batteries.

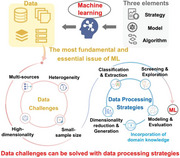

## 1 Introduction

In the realm of power and energy storage, significant progress has already been made in the development of secondary batteries, particularly for lithium batteries. However, lithium batteries still face challenges of unsatisfactory energy density, inferior rate capability, short cycle life, and poor safety.^[^ ^1^ ^]^ From the perspective of materials researchers, these challenges mainly arise from the difficulty in comprehending the relationship between material structures and battery performance at the microscopic level. To investigate these relationships, researchers have conducted a large number of experiments over the past decades. Unfortunately, the subjective nature of human activity easily results in complex and contradiction research findings. Therefore, it is extremely challenging to extract accurate relationships from these experimental results, of which the existing relationships are often based on specific data and lack generality. With the advancement of computer technology, and the refinement of computational modeling and simulation methods, such as first‐principles calculations, density functional theory calculations (DFT), molecular dynamics simulations (MD), Monte Carlo simulations, phase‐field simulations (PFM) and finite element simulations, make it possible to explore the underlying relationships within experimental results.^[^ ^2^ ^]^ These methods enable researchers to establish the underlying relationships within experimental results, including understanding the microphysical mechanisms and predicting the performance of unknown materials, thereby enhancing the effectiveness of experimental guidance.^[^ ^3^ ^]^ Nevertheless, due to the complexity of these relationships, researchers often need to engage in multiple trial‐and‐error attempts before achieving satisfactory results. Furthermore, the high cost associated with theoretical calculations limits the practicality of this approach.^[^ ^4^ ^]^

“Material genomics” is a new research method that aims to accelerate the research and application of new materials, which utilizes key technologies such as efficient computing, high‐throughput experiments, and big data analysis of materials. By constructing three innovation platforms: computing, experiments, and databases, the material genome helps to efficiently and accurately establish potential relationships between structure and performance.^[^ ^5^ ^]^ Corresponding to these three innovation platforms, there are three working modes of the material genome: calculation‐driven, experiment‐driven, and data‐driven. The calculation‐driven mode relies on computational simulations to study potential relationships, predict candidate materials, and narrow down the scope for experiments. In the experiment‐driven mode, high‐throughput synthesis and characterization experiments are used to explore potential relationships, and quickly optimize or screen materials. The data‐driven mode utilizes big data, data mining, and artificial intelligence to explore deeper relationships that promote material design and discovery. Both the calculation‐driven and experiment‐driven modes focus on high‐throughput manual handling and screening. However, the subjective initiative of researchers and limited resources of reality will weaken the interpretability of predicting results. Additionally, these modes normally generate a large amount of scientific data, posing a challenge for researchers to manage and utilize it efficiently. This calls for the introduction of artificial intelligence technologies like machine learning (ML) in the field of materials research. ML, as an objective and data‐driven model, combines the expertise of domain experts with data analysis to uncover the potential relationships hidden in data effectively, which offers advantages in terms of efficiency and result reliability, becoming an emerging direction of material genetic engineering.^[^ ^6^ ^]^

ML has gained significant attention in lithium battery materials research. It contributes to several key areas: enhancing the efficiency of new material development and synthesis,^[^ ^7^ ^]^ generating interatomic potentials that match or exceed DFT accuracy for MD simulations,^[^ ^8^ ^]^ extending high‐fidelity battery state simulations to extreme conditions,^[^ ^9^ ^]^ interpreting materials' physical and chemical properties through identifying highly correlated descriptors,^[^ ^10^ ^]^ constructing new descriptors to elucidate complex material properties.^[^ ^11^ ^]^ and predicting battery lifespan.^[^ ^12^ ^]^ Although ML techniques have demonstrated considerable benefits in enhancing the research and application of lithium battery materials, they typically necessitate high‐quality training data. Researchers often concentrate on improving the accuracy and reliability of ML techniques. However, they frequently overlook a fundamental issue—the data quality issues within ML.

Lithium battery materials data accumulates ceaselessly throughout the entire life cycle of lithium battery material development. Specifically, the data comprises several categories: theoretical calculation data that arises from predictive models, empirical measurement data obtained from laboratory experiments, and model prediction data generated through simulations.^[^ ^13^ ^]^ ML first uses known data to train the model, and then uses the trained model to predict the output of unknown input data. Thus, it is important to note that the reliability of the training results in ML depends on the quality of the data, rather than just the algorithm, strategy, or model used.^[^ ^14^ ^]^ Utilizing advanced techniques to thoroughly analyze the underlying information and relationships within this data can tackle the issues caused by the poor quality of lithium battery materials data. This enables the creation of reliable and precise prediction models, exhibiting high accuracy under particular operational conditions in the lithium battery materials domain.^[^ ^15^ ^]^ Furthermore, data processing plays a significant role in ML, which occupies about 80% of the time in the ML process. Therefore, enhancing data quality to improve the efficiency of ML is crucial, especially for establishing potential relationships and improving overall effects. At present, a systematic compilation of lithium battery material data is lacking, which limits the understanding of the data significance within the realm of lithium battery materials.^[^ ^16^ ^]^

In this review, we initially provided a brief overview of the advantages of ML in exploring the structure‐activity relationships of lithium battery material data. Subsequently, we delved into more intricate and essential data challenges, with the aim to methodically summary the data challenges and address data quality issues by incorporating existing research in the lithium battery material field. The data challenges include multi‐sources, heterogeneity, high dimensionality, and small‐sample size in ML is comprehensively examined in terms of the structure‐activity correlation within lithium battery material data. The effective strategies for addressing data quality issues are outlined, encompassing classification and extraction, screening and exploration, dimensionality reduction and generation, modeling and evaluation, and domain knowledge incorporation, where the data quality, model reliability, and interpretability can be enhanced. In addition, these methods also hold excellent application scenarios in battery classifying and recycling technology, the extraction of multidimensional descriptors from TEM and phase‐field simulation images, the development of new ML models, the generation of time‐series data, and so on. In the end, novel strategies for data management and data analysis, with two main innovative pathways to tackle data challenges will be proposed.

## 2 Brief Overview of ML

The essence of ML lies in the division and classification of feature vectors in a high‐dimensional space. In other words, ML provides a way to identify the relationship between known or controllable inputs (features) and unknown or uncontrollable outputs. This allows ML to handle complex data that may not have direct value to an organization or society, and transform it into knowledge through analysis, leading to reliable and repeatable results.^[^ ^17^ ^]^ From a mathematical theory of view, ML consists of three main elements: the model, the strategy, and the algorithm. The model is a function, which predicts the output when giving the input for a specific problem. The strategy is used to evaluate the quality of the model through expected risk or loss functions. The algorithm is the specific calculation method used to find the optimal model in the model space based on the evaluation criteria.^[^ ^18^ ^]^

**Figure 1:**
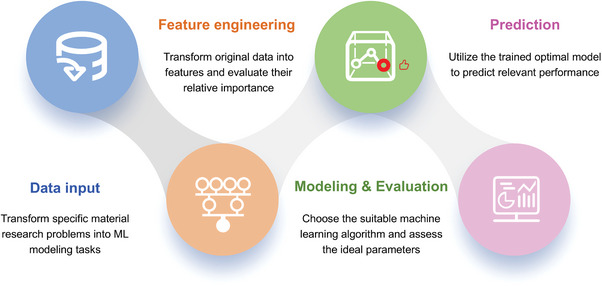
The procedure of ML with data input, feature engineering, model construction and evaluation and its function.

In practical implementation, ML typically involves three key stages including data input, feature engineering, model construction, and evaluation (**Figure** **1**). 1) Data input can be divided into target definition, data preparation, and data preprocessing. The target definition aims to transform specific material research problems into ML modeling tasks, which are driven by the specialized theory‐knowledge in the particular discipline or field (domain knowledge). Data preparation represents obtaining data through experiments, calculations or searching existing databases, extracting appropriate descriptors, and establishing corresponding data sets. Descriptors refer to a set of data or data structures associated with the “described object”, which are mostly used to record the attributes of the described object. Data preprocessing refers to the process of cleaning (removing missing values, duplicate values, and outliers), transforming (logarithmic transformation), and normalization (standardization and regularization) raw data based on specific tasks and models before conducting data analysis and modeling, which is intended to improve data quality before model training through a series of methods. 2) Feature engineering is the process of transforming the original data into features that better express the essence of the problem. The application of these features to the prediction model can improve the prediction accuracy of the data model. Feature engineering mainly includes feature transformation and feature selection, where feature transformation refers to mapping high‐dimensional feature space to low‐dimensional feature space, and feature selection refers to selecting a feature subset from all feature species. 3) The model construction refers to selecting the appropriate ML algorithm and adjusting the optimal parameters, with the purpose to simulate the mapping relationship between the condition attributes and target attributes. Model evaluation aims to evaluate the quality of a model in terms of performance, computing resources, and interpretability, that is, whether it meets the goal specified by the learning task.^[^ ^19^ ^]^

In the domain of lithium battery technology, ML holds significant importance for implementation in three key areas. At the system level, ML refines battery charging and discharging strategies. It enables real‐time monitoring and estimation of the battery's state of charge (SOC) and state of health (SOH), which enhances the overall performance and reliability of the battery system. Additionally, machine learning accurately predicts the remaining lifespan and performance degradation of lithium batteries, which is vital for ensuring stable equipment operation and preventing accidents. At the battery level, ML could optimize battery design and structure, thereby enhancing the intelligence and efficiency of manufacturing processes. It fosters interdisciplinary collaboration, such as chemistry, physics, and computer science, which promotes the innovative research of lithium batteries. And from the viewpoint of the material hierarchy primarily examined in this article, ML techniques could efficiently process and analyze extensive experimental and computational datasets, as previously emphasized, ML aslo offers significant benefits in exploring the relationship between the materials structure and battery performance at the micro level,^[^ ^20^ ^]^ of which these relationships are crucial for understanding and enhancing the performance of lithium battery materials. For example, one key factor that affects metal‐ion battery performance is the volume change of the graphite anode during the charge and discharge processes, which is caused by the continuous (de)‐intercalation of metal ions. Since this volume change results in considerable destroy of the graphite anode, it has a large impact on the charge and discharge capabilities.^[^ ^21^ ^]^ In a study conducted by Moses et al., they collected a large amount of data from a database, including information on metal ion types, electrode reaction types, Bravais lattice types, space groups, average voltage, and electrode volume change percentage. Using the Matminer software, they generated additional atomic features and built a deep neural network model, which successfully established the relationship between the volume change caused by the (de)‐intercalation of metal ions and the average voltage during battery operation. Through further cross‐validation and independent tests, they provided a basis for predicting this battery performance.^[^ ^22^ ^]^ Furthermore, during the charging process of a lithium metal battery, the negative potential continues to decrease, causing more charges to accumulate at the tip of the electrode surface. Under certain conditions, lithium ions will deposit at the tip and form dendrites, which may lead to internal short circuits and result in thermal runaway, fire, and even explosion. Therefore, it is crucial to explore the relationship between the adsorption–migration process of ions and the growth of dendrites.^[^ ^23^ ^]^ Ma et al. conducted an artificial neural network model to investigate the correlation between the growth of lithium dendrites and the surface microstructure of lithium metal, as well as the physicochemical parameters of the liquid electrolytes. They established a relationship between Li^+^ concentration, its concentration gradient at the lithium/electrolyte interface, and lithium dendrite growth.^[^ ^24^ ^]^ Before metal ions are embedded in the electrode, they form a solvated structure by complexing with the solvent. This solvated structure is then transported to the electrode surface, where a de‐solvation process occurs that involves the reaction and transport of ions on the electrode. By studying the solvation structure and de‐solvation process of Li^+^, researchers have made attempt to establish a structure‐activity relationship between solvation structure, de‐solvation behavior, and electrochemical performance, with the aim to improve the rate performance and stability of batteries.^[^ ^25^ ^]^ Wang et al. proposed a computational method that combines ML with molecular dynamics to study the solvated structure of an electrolyte based on trigylme. They calculated the densities of electrolytes at different concentrations by using ML molecular dynamics (MLMD) and found good agreement with experimental results.^[^ ^26^ ^]^ Overall, ML will greatly accelerate the material screening process and enhance the prediction of battery performance across various conditions. By extracting valuable insights from data, ML empowers researchers to make informed scientific decisions, ultimately improving the energy density and safety of batteries. Despite notable progresses have been made in utilizing ML for lithium battery materials, the comprehension of ML techniques in this field is still in a very preliminary stage. Specifically, our grasp of essential knowledge regarding electrochemical processes of battery operation, and the accuracy of the data and model is nascent. To facilitate the development of lithium battery materials, systematic overview and research on the datasets employed in ML is crucial.

## 3 Data Challenges of Lithium Battery Materials

In the domain of lithium batteries, data quality signifies the caliber of battery data accessible to testers. This quality is typically assessed through criteria such as accuracy, completeness, consistency, reliability, and effectiveness. While numerous definitions exist, data is deemed high‐quality if it fulfills its intended applications in lithium battery development, model decision‐making, and system monitoring.^[^ ^27^ ^]^ High‐quality data should exhibit six essential characteristics: 1) Accuracy. The data must accurately reflect both the macroscopic and microscopic structures, physical and chemical properties, and the evolution of electrochemical processes in lithium batteries. Furthermore, the data source must be verifiable and credible. 2) Consistency. The data should remain uniform across various battery testing systems and feature sets, with no discrepancies in identical feature values across different systems or datasets in lithium batteries. 3) Validity. The data must adhere to the rules and parameters established by foundational theories in lithium battery research, ensuring the correctness of its structure, the physical and chemical relevance of its values, and the inclusion of accurate values. 4) Completeness. The dataset should encompass all anticipated values and data types, including any feature data pertinent to the lithium battery dataset. 5) Timeliness. The data must be current, accurately reflecting the operational status and future trends of lithium batteries, thus being readily available when required. 6) Uniqueness. Each record within a single lithium battery dataset must be distinct, with no duplicate entry, allowing for unique identification of each record.^[^ ^28^ ^]^

A battery dataset that satisfies these criteria proves more reliable and trustworthy than one that does not. However, experimenters may also evaluate datasets based on additional qualities such as correlation, reliability, and repeatability, drawing from established knowledge in physics and chemistry. The overarching aim is to ensure that the data serves its intended purpose and can be relied upon. Maintaining high data quality enables organizations to reduce the costs associated with identifying and fixing bad data when a data‐related issue arises. In addition, high data quality increases the accuracy of analytics, leading to better ML decisions, which in turn can promote the screening and development of battery materials, prediction of battery performance, formulation optimization, health monitoring, and other technologies.

The lithium battery materials data is always multi‐sources, which makes data processing time‐consuming and energy‐intensive, and may also influence the results of model training. This problem arises from variations in data generation, collection, and recording methods. The main sources of lithium battery materials data include experimental databases like CSD, ICSD, Pauling file, NOMAD, NIST,^[^ ^29^ ^]^ and computational databases like Materials Project, AFLOWLIB, OQMD, Mat Cloud, and so on.^[^ ^30^ ^]^ Additionally, the data collected by crawlers in the literatures and the data obtained by enterprises' own experiments are also regarded as effective sources of lithium battery materials data.^[^ ^31^ ^]^ However, there may have inconsistencies in attribute values within a single entity or between different paired entities in these databases.^[^ ^32^ ^]^ Besides, when using multiple databases to provide attribute values for the same entity, conflicts will arise.^[^ ^33^ ^]^ Therefore, addressing the challenge of multi‐sources in lithium battery materials data is crucial.

The heterogeneity of data refers to the presence of incomplete data, noise, and structural differences. For ML, samples are often obtained by combining data from multi‐sources, such as from the subgroups at different times or using different technologies. Each subgroup may have unique characteristics that other subgroups do not possess. Data points from small populations with small‐sample sizes are often considered as “outliers” or noise. Additionally, due to differences in data structure among subgroups, various data loss and gaps during data acquisition may exist. These incomplete data may cause issues with data quality, such as mismatch with ML modeling requirements, which can affect the reliability of ML models.^[^ ^34^ ^]^ Data incompleteness denotes the existence of absent values or insufficiently gathered data during the training and testing stages of ML frameworks. This complication may arise from multiple factors, including inaccuracies in data collection methodologies, sensor failures, user interactions, or data privacy regulations. Such incompleteness detrimentally affects model performance and efficiency, resulting in biased forecasts and unreliable outputs, which is a facet of data heterogeneity. In the realm of lithium battery research and applications, time series data pertains to information accrued over time, which was primarily utilized to assess battery functionality, health status, and charge–discharge characteristics. While time series data holds rich content, it frequently encounters problems such as missing values, anomalies, and uneven distributions, which often link to sensor malfunctions or data gathering errors. This data typically appears in an unstructured form, with timestamps potentially mixed and not arranged correctly. Moreover, in many instances, the date‐time column defaults to a string format, requiring conversion to a date‐time format prior to any analytical processes. These elements exemplify the hurdles tied to data heterogeneity.

The concept of high dimensionality in data refers to the situation where data sets contain a large number of descriptors, particularly when integrating data from various sources, which leads to challenges such as data sparsity and redundancy, resulting in excessively high data dimensions. Data sparsity occurs when the number of variables or parameters is much higher than the sample size. Data redundancy refers to that many of the numerous attributes in the data are unrelated to the ongoing task. This high dimensionality can lead to accidental endogeneity, where many uncorrelated covariates may coincidentally relate to the residual noise. Consequently, the endogeneity will introduce statistical bias, leading to inconsistencies of model selection and errors. In addition, high dimensionality can introduce false correlations between the response variable and unrelated covariates, potentially leading to incorrect statistical inferences or erroneous scientific conclusions, which is not conducive to effectively uncover the underlying or general patterns in the data by using ML models.^[^ ^35^ ^]^

**Figure 2:**
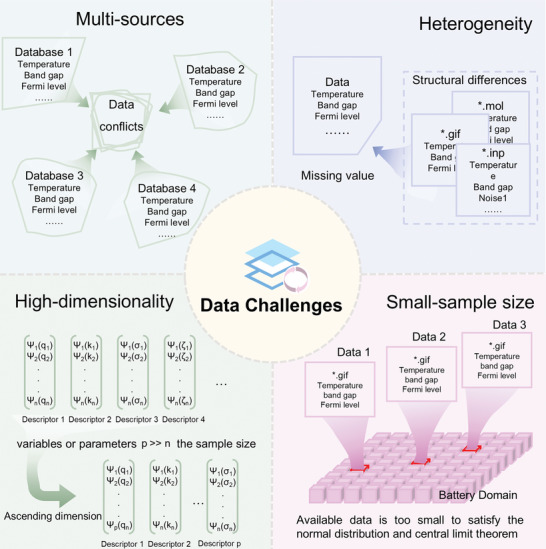
Existing data challenges of materials in the battery field.

The efficacy of any classification model is contingent upon whether it meets certain criteria with the data sets utilized during both training and testing. ML, when trained on large‐scale data, can effectively train and test models to make accurate predictions. Specifically, a larger, well‐balanced, and more representative data set bestows greater certainty in the model's effectiveness and subsequent outcomes. Regrettably, extensive data sets are generally unavailable to the researches within the realm of battery applications, resulting in limited sample sizes for lithium battery materials data. Small‐sample of data refers to a situation where the sample size is too small to meet certain assumptions, such as normal distribution and central limit theorem. The lack of sufficient data often results in under fitting or over fitting of models, compromising their accuracy.^[^ ^36^ ^]^ To sum up, because of the complex nature of lithium battery material data, when dealing with ML, there are data challenges including multi‐sources, heterogeneity, high dimensionality, and small sample sizes, as represented in **Figure** **2**.

This article categorizes data into structured and unstructured data. In the battery sector, structured data refers to information regarding battery performance, specifications, and operational parameters that are recorded in a uniform format, making it quantifiable and analyzable. This data typically resides in databases for real‐time monitoring and management. Each record contains identical fields, such as battery model, capacity, charge/discharge cycle, internal resistance, and temperature, which facilitates analysis. Unstructured data describes information that cannot be cataloged in a uniform manner. This type of data often encompasses various documents, images, and other forms of information related to battery use and development.^[^ ^37^ ^]^ Unstructured data can take many forms, including text, images, and videos, which covers a broad spectrum of information from experimental records to user feedbacks. Structured data mainly serves to quantify and monitor battery performance and status for analysis and decision‐making, while unstructured data provides additional information, aiding in research and advancements in battery technology. The integration of both types of data can enhance the efficiency of battery management and development. The data processing methods outlined in this article aim to address the handling of structured data represented by numbers and unstructured data represented by images.

## 4 Solutions to Data Challenges of Lithium Battery Materials

In the succeeding section, we will elaborate on the four most crucial techniques of ML, namely, classification and feature extraction, screening and exploration, dimensionality reduction and generation, modeling, and evaluation, to address the data challenges of multi‐sources, heterogeneity, high dimensionality, and small sample sizes. It is important to emphasize that each technique comprises a range of technologies and is not specifically tailored to address a singular data challenge. By leveraging the core principles based on these four techniques, multiple data problems can be addressed concurrently. Meanwhile, we believe that the integration of these methods and ML can offer solutions for key challenges within relevant domains. On the other hand, the interpretability of ML outcomes in the field of lithium battery materials is subjected to some degree of randomness, of which this uncertainty has led researchers to question the reliability of data transmission and the rationale behind model construction. As highlighted by Cui et al., this is due to the fact that data‐driven ML heavily relies on the quality of the sample data and lacks guidance from domain expertise.^[^ ^36a^ ^]^ Therefore, the importance of incorporating domain knowledge into data problem‐solving will be further emphasized.

### 4.1 Classification and Extraction—A Solution to the Data Issue of Multi‐Sources

**Figure 3:**
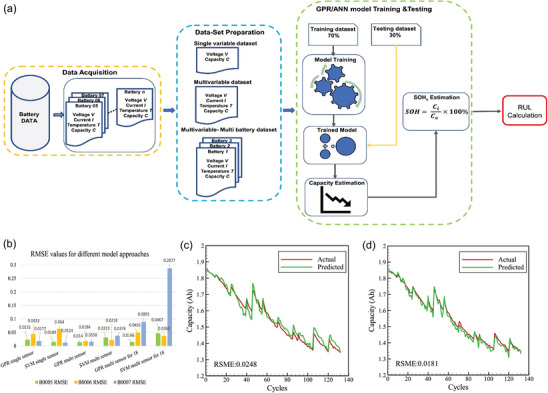
a) Schematic illustration of multi‐sources data acquisition, training data sets, and capacity estimation with GPR and SVM models. b) Comparative histograms for RMSE values against single dataset and multi dataset. GPR model‐based actual versus prediction plots for c) single‐sensor datasets and d) multi sensor datasets. Reproduced with permission from ref. [31] Copyright 2021 Elsevier.

Classifying data from various sources and extracting data relevant to the target attributes, particularly descriptors, constitutes an effective approach to address the challenge of multi‐sources data. Howbeit, the intricate nature of lithium battery materials data originated from multiple sources is not conducive for ML modeling. Researchers must process this data in a manner that enables the mapping of relationships between different samples (descriptor and target attribute). This processing aids in screening and standardizing the data, enhancing the convergence of model parameters during training, and reducing data complexity, which is a core concept in feature engineering. After classifying different data sets, important features are extracted and multi‐source data fusion strategy is carried out. This approach combines data from various origins to enhance our comprehension of battery performance, condition, and health. Such fusion increases the model's accuracy and robustness while unveiling potential features and relationships. Nagulapati et al. proposed a combined multi‐data‐based method to improve the prediction accuracy, which clearly reflects the advantages of multi‐sources dataset compared to the single dataset, as specific illustrated in **Figure** **3**.^[^ ^31^ ^]^ The extraction of descriptors for multi‐source spatial data presents opportunities for analyzing the issue from various perspectives. Descriptors, being a crucial component of material data, directly influence the prediction accuracy and model reliability. By identifying descriptors determining the specific properties or functions of materials, the adverse effect of multi‐source data in ML can be mitigated.^[^ ^38^ ^]^

**Figure 4:**
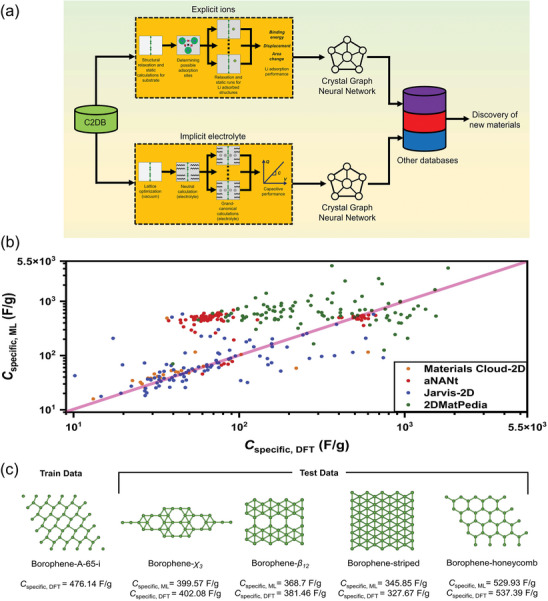
a) Schematic illustration of the high‐throughput workflow combining DFT and ML to choice the descriptors refer to multi‐sources database. b) Data analysis and performance of the ML model for LIB application. c) Discovery of new materials from other databases using ML. Reproduced with permission from ref. [41] Copyright 2022 Elsevier.

Descriptors can be classified into structured and unstructured descriptors. Thus, one feasible approach is to convert multi‐sources materials data into structured descriptors required by ML models. Through screening from various literatures, Gharagheizi et al. created a database that contains 977 experimental data points for 54 ionic liquids. Data preprocessing eliminates questionable data. K‐means clustering then identifies the training set, validation set, and test set. A novel validation technique *R*_m_^2^ metrics has been extensively used to confirm the validity of the developed model. They employed a sequential search algorithm and quantitative structure‐property relationship (QSPR) method to identify the optimal set of molecular descriptors, which includes temperature, four descriptors based on anions, and five descriptors based on cations. Using these selected parameters, they generated a least square support vector machine‐quantitative structure‐property relationship (LSSVM‐QSPR) method. The proposed model exhibited a low average absolute relative deviation of less than 1.9%.^[^ ^39^ ^]^ Shi et al. combined quantum computing with ML methods and used 13 intrinsic properties of organic solvent small molecules in a multi‐sources database as descriptors. They constructed a model for disordered/amorphous discharge products and found that the oxygen evolution reaction had a faster rate compared to that of crystalline structures. They also pointed out that the phosphate‐based solvents could promote these decomposition kinetics.^[^ ^40^ ^]^ Kabiraj et al. developed an automated computational framework to evaluate thousands of two‐dimensional materials (**Figure** **4**). Four descriptors were established for the purpose of mapping the adsorption of lithium ions from the C2DB database, which enables the exploration and screening of potential candidates efficiently. And the framework has also been demonstrated to be applicable to other four databases. Additionally, ab ML model based on crystal graphs was devised to expedite the discovery of materials for lithium‐ion batteries and supercapacitors.^[^ ^41^ ^]^ To improve the reusability of descriptors, researchers have developed toolkits to integrate the existing descriptor extraction methods. For example, Matminer is an open‐source software platform based on Python that facilitates data‐driven methods for analyzing and predicting material properties, which utilizes various feature extraction modules to generate descriptors related to physical quantities, reducing the difficulty of selecting descriptors.^[^ ^42^ ^]^ Another toolkit, DScribe, created by Himanen et al., focuses on atomic property prediction using ML, which can encode atomic structures and descriptors, such as Coulomb matrix, Ewald sum matrix, sine matrix, many‐body tensor representation, atom‐centered symmetry functions, and position‐smoothed overlaps. DScribe demonstrates its applicability by accurately predicting atomic ion charges in organic molecules.^[^ ^43^ ^]^

In the domain of batteries, features involve not only the structural specifics of different‐sources molecules but also the aspects of chemical properties. By assessing the electrostatic interplay between Li+ and solvent, he et al. computed the electrostatic potential (ESP) of the electrolyte solvent through DFT. They utilized ESP as a straightforward solvent characteristic, effectively identifying potential soluble solvents, feeble soluble solvents, and antisolvents in electrolytes via the ESPmin–ESPmax chart. This can serve as a critical guideline to promote the development of high‐performance electrolytes.^[^ ^11^ ^]^ Wang et al. discovered that the donor number (DN) serves as a key parameter for designing the local high‐concentration electrolytes (LHCEs) for LIBs. Specifically, the primarily solvation solvent should possess a DN > 10, while the diluent should have a DN ≤ 10. Through a combination of experimental and theoretical calculations, it has been demonstrated that the DN value of the solvent is a dependable measure of its coordination capability with Li^+^.^[^ ^44^ ^]^ Fan et al. extracted the molecular radius (*r*_s_) and Li^+^ transport energy barrier (*E*_trans_) as descriptors from multiple solvent molecular datasets, which allowed them to identify the fluoroacetonitrile (FAN) as the optimal solvent molecule. The computational analysis and experimental validation demonstrated that using FAN as an electrolyte solvent is able to endow the LIBs with high energy density, rapid charging capability, and a broad operating temperature range simultaneously.^[^ ^45^ ^]^

Using deep learning techniques, we have the ability to automatically extract and analyze information from various sources of unstructured data, such as images and graphics. By extracting unstructured descriptors, it is possible to bypass the traditional process of feature engineering in ML and achieve accurate predictions of material properties. In the field of graph data, Xie et al. have developed a crystal graph with a convolutional neural network framework that can directly learn material properties from the atomic connections in crystals. This framework overcomes the conventional challenge of multi‐sources of material data and provides a universal and interpretable representation of crystal materials, which accurately predicts various properties, such as formation energy, band gap, Fermi level, and elastic modulus of crystals. Based on these predictions, they performed computational screening on over 12000 inorganic solids and predicted over 20 mechanical anisotropy interfaces that can suppress the dendrite growth.^[^ ^46^ ^]^

**Figure 5:**
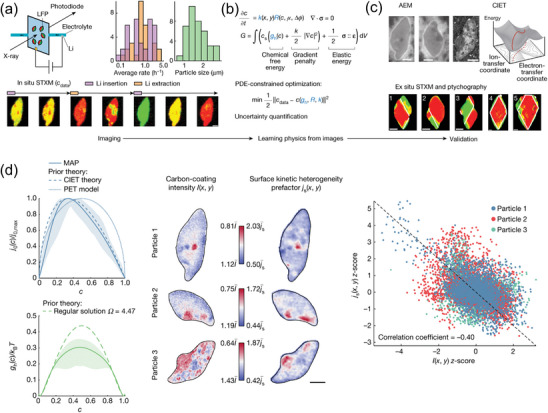
a) Using in‐situ STXM, spatiotemporal mapping of lithium concentration in LFP nanoparticles averaged of 39 particles in the [010] direction during battery cycling. Histograms of the particle size (major axis length) and average charge and discharge rates of all imaged particles. b) In situ STXM images are used as training data in the optimization, which minimizes the squared sum of the errors at all the pixels subjected to the constraint of the model, with respect to the unknowns—homogeneous free energy g_h_ c), reaction rate R and spatial heterogeneity k (x, y)—highlighted in blue. Cross‐validation and uncertainty quantification are performed subsequently. c) The heterogeneity k (x, y) is further validated using AEM, the mechanical model with the morphology of the phase‐separated domains from ex‐situ STXM (particles 1–3) and ptychography images (particles 4 and 5) of fully relaxed particles, and the reaction kinetics with CIET theory. d) Comparison of the constitutive laws of reaction kinetics and thermodynamics obtained from image inversion with theoretical models, the AEM carbon signal I (x, y) and inferred spatial variation of surface reaction rate *j*_s_ (x, y), the rescaled j_s_ (x, y) and AEM image intensity I (x, y) for the three particles. Reproduced with permission from ref. [48] Copyright 2023 Nature.

For the image data, Dixit et al. have adopted a deep convolutional neural network based on ResNet‐34 to segment lithium metal and pores in X‐ray tomography images of lithium metal from different sources. This enables quantitative tracking of the morphological changes at the interfaces between the lithium metal electrode and the solid‐state electrolyte during cycling. Microscopic simulations show that regions with lower effective properties (i.e., transport and mechanical properties) tend to be attacked.^[^ ^47^ ^]^ Using ML techniques, Zhao et al. conducted an experimental study to map the regions of instability in the free energy landscape, which are typically challenging to investigate. They combined a large dataset of scanning transmission X‐ray microscopy (STXM) images with electrochemical phase‐field models that adhere to thermodynamic principles. To achieve this, they employed techniques such as partial differential equation (PDE) constrained optimization, UQ, parameterize the unknown homogeneous free energy g_h_(c), and exchange current j_0_(c) and cross‐validation. Through these methods, they obtained valuable insights into the free energy landscape and reaction kinetics, which were found to be consistent with existing theoretical models of coupled ion‐electron transfer (CIET), and were able to explain the spatial variations in reaction rates. The results of their study demonstrated that the simulated images generated from the learned model were nearly identical to the experimental images. By implementing systematic regularization techniques, the training error decreased to 6.8%, while the validation error measured 9.6 ± 0.9%, in comparison to the initial model. By leveraging advancements in scientific ML and UQ, Zhao et al. have developed a pixel‐wise image inversion approach that combines data‐driven insights with physics‐based knowledge. This approach shows great potential to be applied in various fields, including but not limited to energy materials, soft matter, and biology, which offers an alternative approach to non‐destructive imaging and enables the learning of constitutive rules from rich image data. The detailed procedure is illustrated in **Figure** **5**.^[^ ^48^ ^]^

On the other hand, the data volume is vast and varied, leading to a range of diverse models. Efficient classification and utilization of these models are crucial, especially in resolving conflicts from various data sources. Models can be categorized based on factors like linearity and learning methods. Models within the same category can be merged as sub‐models to enhance overall performance, namely model‐fusion, which not only improves model performance but also reduces training costs, reflecting the future trend of development.^[^ ^49^ ^]^ To address conflicts in merging different model types, Stoica et al. introduced a universal method, “ZipIt!”, to combine two arbitrary models of the same architecture with two simple strategies. This approach involves extending the model merging problem to accommodate feature merging within each model, along with incorporating compression models to support specific layers, which eventually creates a multi‐head model. These modifications led to a remarkable improvement of 20–60% in performance over previous methods, enabling the merging of models trained on distinct tasks.^[^ ^50^ ^]^ Employing cluster analysis to categorize data reveals natural groups or clusters in the feature space and endows a deeper comprehension of the internal structure and patterns within the problem domain. This insight uncovers hidden patterns and correlations in the data, serving as guidance for feature engineering and descriptor extraction.^[^ ^51^ ^]^ Similarly, various classification algorithms like logistic regression (LR), decision trees (DR), random forests (RF), support vector machines (SVM), k‐nearest neighbor algorithm (k‐NN), artificial neural networks (ANN), and AdaBoost can categorize data into different groups, which facilitates the comprehension of data relationships and patterns, revealing crucial distinguishing features among categories.^[^ ^52^ ^]^

### 4.2 Screening and Exploration—A Solution to Address the Heterogeneous Data

Screening and exploration represent fundamental technologies in data structure management. They primarily concentrate on filtering and processing anomalous data, selecting a standardized data format that is beneficial for ML, organizing data structures, and investigating potential correlations. These methods enhance both the credibility and interpretability of the data, subsequently enhancing its reliability. Ultimately, high‐quality data can significantly enhance the practical applicability of ML models and facilitate the accurate exploration of the relationship between material structure and performance. Standard screening techniques encompass handling missing values, refining noisy data, detecting and processing outliers, encoding and characterizing data, identifying outliers, addressing uneven data distribution, filtering feature selection,^[^ ^53^ ^]^ sequential search algorithm,^[^ ^39^ ^]^ and more.

**Figure 6:**
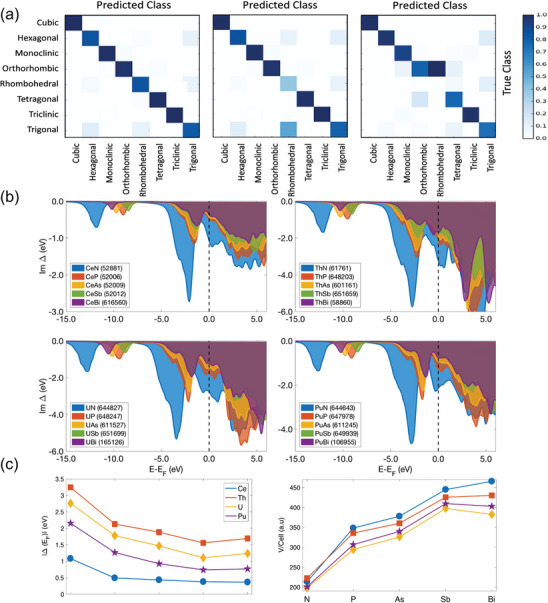
a) Confusion matrices for different ML algorithms of data preprocessing. b) Trends in f‐orbital hybridization function. The calculated hybridization function Δ(E) for the series of 4f and 5f monopnictides, LnX and AnX (Ln =  Ce, An = Th, U, Pu, and X = N, P, As, Sb, Bi). c) Volume versus hybridization function at the Fermi level. The value of hybridization function at the Fermi level, Δ(*E*_F_) (top), and the volume per unit cell (bottom) for the series of monopnictides considered in (b), showing an anti‐correlation between the degree of localization and lattice constant. Reproduced with permission from ref.[54] Copyright 2018 Nature.

Hasnain et al. collected compound structure files containing lanthanide and actinide elements from two databases, namely the Inorganic Crystal Structure Database (ICSD) and Crystallography Open Database (COD), which has 54465 and 27502 compounds, respectively. They utilized three supervised ML algorithms to perform the “data cleaning” task, which involves evaluating the accuracy, precision, and recall of the predictions using 10‐fold cross‐validation. They created a confusion matrix to visualize the errors between the predicted and actual class results. Subsequently, they proposed and implemented a data‐driven approach to assist in the materials discovery process by deploying state‐of‐the‐art algorithms and query tools, as demonstrated in **Figure** **6**. Through the utilization of this algorithm, they successfully purified and extracted dependable structural information from the database, effectively resolving uncertainties due to missing information in CIF files and correcting the structure files necessary for producing reliable electronic structure data.^[^ ^54^ ^]^ Another study by Charagheizi et al. used the Least Squares Support Vector Machine (LSSVM) to characterize and predict the conductivity of ionic liquids. They manually verified the heterogeneous conductivity data obtained from experimental measurements and calculated molecular descriptors. By optimizing the temperature and descriptors using a sequential search algorithm and excluding suspicious data points, they achieved an average prediction bias of only 1.9%.^[^ ^39^ ^]^ In particular, Draxl has successfully introduced the FAIR principles to data‐driven materials science research. She advocated the creation of repositories, standardized data archives, encyclopedias of data knowledge, advanced analysis of big data, and visualization tools to ensure the accuracy and reliability of material data.^[^ ^38a^ ^]^

As the fundamental and underlying pillar of ML, the significance of data is indisputable. Besides the explicit linkages within data, delving into its implicit associations is crucial. Prior to investigation, comprehending the data thoroughly is essential, encompassing both external and internal information. External data can be regarded as practical importance with the value of data within its relevant domain. Integrating field‐specific information aids in better comprehension and manipulation of data, such as the data processing technique of embedding domain knowledge. Internal data refers to intrinsic data characteristics, such as mean, standard deviation, kurtosis, skewness, standardization, discretization, and regularization. Data standardization serves as a critical approach for ensuring consistency and comparability in data, particularly when dealing with varied experimental devices. This practice helps to mitigate biases, curtail noise, and enhance the efficiency of training and predictive accuracy of models. When predicting the electrochemical properties of lithium batteries, data standardization transforms feature data to achieve a mean of 0 and a standard deviation of 1, aligning it with a standard normal distribution. This transformation lessens the impact of disparate feature scales and boosts the model training stability. In the analysis of charge and discharge data, normalizing current and voltage allows for more clear comparisons of battery performance across different experimental contexts. Analysis techniques include value counts function for discrete data, covariance analysis for continuous data, Pearson correlation coefficient analysis, spearman correlation coefficient analysis, and uncertainty quantification, among others. Uncertainty Quantification (UQ) stands as a critical domain within science and engineering. It centers on the identification, analysis, and characterization of uncertainties presented in models, data, and system behaviors. The primary objective is to assess and manage these uncertainties through quantitative approaches, thus enhancing the precision and dependability of predictions. Deng et al. successfully quantified the uncertainties related to model parameters and predictions by employing Bayesian statistical methods. They constructed a sparse Gaussian Process Regression (GPR) model to estimate battery SOH, using two proposed features and the mean of voltage segments as inputs. They compared the performance of their model against other typical data‐driven methods, including Multiple Linear Regression, Support Vector Machines, Relevance Vector Machines, and Convolutional Neural Networks. Among these, the sparse GPR achieved the highest estimation accuracy, with an average absolute error of only 1.51%.^[^ ^55^ ^]^

**Figure 7:**
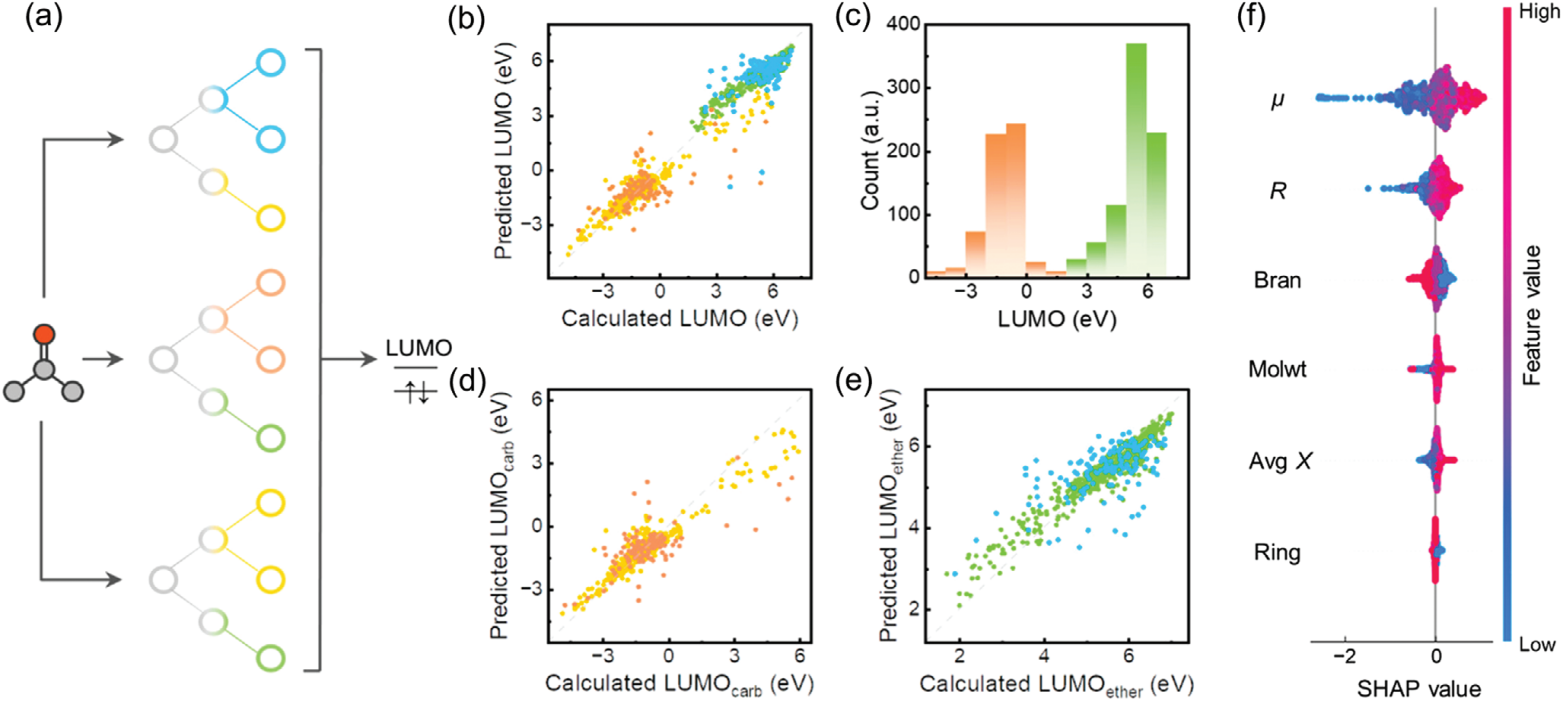
Prediction and interpretation of ML for LUMO energy levels of ion–solvent complexes. a) Schematic diagram of the random forest (RF) model. b) Results of RF model prediction for all 1399 molecules. c) Distribution of LUMO energy levels of ion–solvent complexes. Carbonyl compounds and ethers are mainly included in the red and green bars, respectively. d) Results of RF model prediction for carbonyl compounds. e) Results of RF model prediction for ethers. The training set is marked with yellow or green. The test set is marked with red or blue. f) Shapley features ranking for ethers. SHAP value means the contribution of the sample point to the model's performance. Reproduced with permission from ref. [51] Copyright 2023 ACS Publications.

Exploring the potential connections within data and selecting crucial descriptors or features stands is of vital importance for addressing data challenges, which simultaneously opens up a significant avenue in the realm of battery research. Zhang et al. employed graph theory‐based algorithms to create an extensive database of potential solvent molecules. They represented solvent molecules as graphs, where atoms (carbon and oxygen) served as vertices and bonds (single and double) functioned as edges. Given the prevalent application of ethers and carbonyl compounds as electrolyte solvents, the algorithms initiated molecule generation from two fundamental compounds (formaldehyde and dimethyl ether), progressively adding vertices and edges. This process continued until the resulting molecule encompassed nine heavy atoms, including carbon and oxygen. Molecules that contain active hydrogen atoms, such as alcohols and acids, were omitted from the generation process. The resulting database includes a total of 1399 solvent molecules, effectively addressing the challenge of limited sample size in lithium battery research. Moreover, they utilized the Pearson correlation coefficient to identify potential features that are strongly correlated with the energy level of the target‐Lowest unoccupied molecular orbital (LUMO), where the number of carbonyl oxygen (#(═O)), the dipole moment (μ), the molecular radius (R), molecular weight (Molwt), the number of rings (Ring), average electronegativity (Avg X), the number of branches (Bran), the ratio of carbon atoms to oxygen atoms (#C/#(═O)), and average ionization energy (Avg I) were ultimately chosen as molecular feature descriptors. By integrating SHAP with the trained model to enhance its interpretability, it was discovered that μ has the most significant impact on the LUMO energy level. Through domain expertise, an exploration of μ revealed a Spearman value of 0.53 (exceeding 0.3), indicating a positive correlation between these two properties. As such, μ emerges as a crucial descriptor for characterizing the reduction stability of electrolyte solvent (**Figure** **7**).^[^ ^51^ ^]^

In addition, some quantifiable/verifiable descriptors/values can be used to explore and evaluate the high data quality of lithium batteries, such as the Interquartile Range (IQR) method identifies outliers. By computing IQR values, analysts can pinpoint and exclude outliers, facilitating the determination of data points for future analysis and therefore enhancing overall data quality. Qiu et al. introduced a fault prediction strategy utilizing the interquartile range (IQR) method to categorize voltage anomalies in lithium battery data. They employed a nonlinear autoregressive exogenous neural network for precise voltage forecasting, enabling swift identification of abnormal voltage data. This approach facilitates accurate diagnosis of battery faults and precise localization of defective batteries, demonstrating robust reliability. By screening abnormal battery data, this method also enhances model training and boosts prediction accuracy.^[^ ^56^ ^]^ Cho et al. developed a fire risk assessment method for lithium battery packs by applying IQR filters to real‐time data derived from electrical measurement factors, which is able to identify significant internal resistance fluctuations during operation. The feasibility of this approach was validated through 8000‐cycle aging tests conducted on a prototype battery module under railway vehicle driving conditions.^[^ ^57^ ^]^ Covariance analysis leverages the covariance matrix to explore feature relationships, allowing for insights into data structures and supporting dimensionality reduction techniques, such as Principal Component Analysis (PCA), to gauge data quality. Xia et al. employed principal component analysis (PCA) to scrutinize lithium battery data. They applied dimensionality reduction and linear transformation techniques to convert multiple features into several independent comprehensive features. A correlation analysis was performed using the covariance matrix. They calculated the contribution rate of each feature, ranked the principal components by their contribution rates, and selected the three most significant new features. The physical implications of the principal components were elucidated based on their eigenvalues and eigenvectors. Eigenvector t_1_ primarily correlates positively with the battery's impedance characteristics while negatively correlating with mass, volume, and capacity. Thus, *t*_1_ serves as the principal component indicative of the battery's impedance characteristics. Similarly, vector *t*_2_ reflects the battery's appearance parameters, while eigenvector t_3_ predominantly represents the temperature parameter of electricity, which is crucial for battery safety and thermal management. By leveraging these three new features, consistency in the charging status across different battery packs has been successfully achieved.^[^ ^58^ ^]^ Z‐score standardization transforms eigenvalues into values with a zero mean and unit standard deviation. The standardized Z‐score serves as a novel feature value, providing a basis for assessing data quality. This approach aids model convergence, particularly in distance‐based models, such as k‐nearest neighbor algorithms. Wu et al. optimized the Z‐score analysis method by replacing the average value in the Z‐score definition with the Hausdorff distance between the voltage curve and the median voltage curve. This adjustment amplifies the differences between normal and defective battery data while avoiding the threshold selection issue in battery data. It aids in identifying anomalous units within battery data, making it more suitable for battery fault detection. The algorithm's effectiveness received further validation with real data and was compared against Threshold methods, Pearson correlation methods, and Shannon entropy weighting methods. The results indicate that the optimized Z‐score analysis method not only demonstrates high reliability but also achieves commendable outcomes without relying on a model.^[^ ^59^ ^]^ Additionally, single‐hot encoding converts categorical variables into numerical formats, making them compatible with ML models. The resulting binary indicator variables derived from single‐hot encoding can effectively function as feature values for evaluating data quality, ultimately enhancing model training effectiveness. The unstructured encoding of complex materials based on chemical composition can expedite the discovery and design of battery materials occupying combinatorial chemical space. Zhuang et al. revealed how unstructured encoding can accurately predict the categories of material compounds for battery applications without the need for time‐consuming measurements of bonding networks, lattice structures, or densities. Experiments confirmed that Mendeleev encoding provides the best balance between model complexity and performance. This encoding decomposes chemical formulas into features representing the concentrations of elements in the periodic table, which effectively supports the binary and multi‐class classification of highly complex material compounds using Logit models, decision trees, and support vector machines, exhibiting superior consistency and interpretability. Even with imbalanced classes, the scores for LR remain above 95%, with DT and SVM achieving scores of 98% and 99%, respectively.^[^ ^60^ ^]^

### 4.3 Dimensionality Reduction and Generation—A Solution for Analyzing High‐Dimensional Data

**Figure 8:**
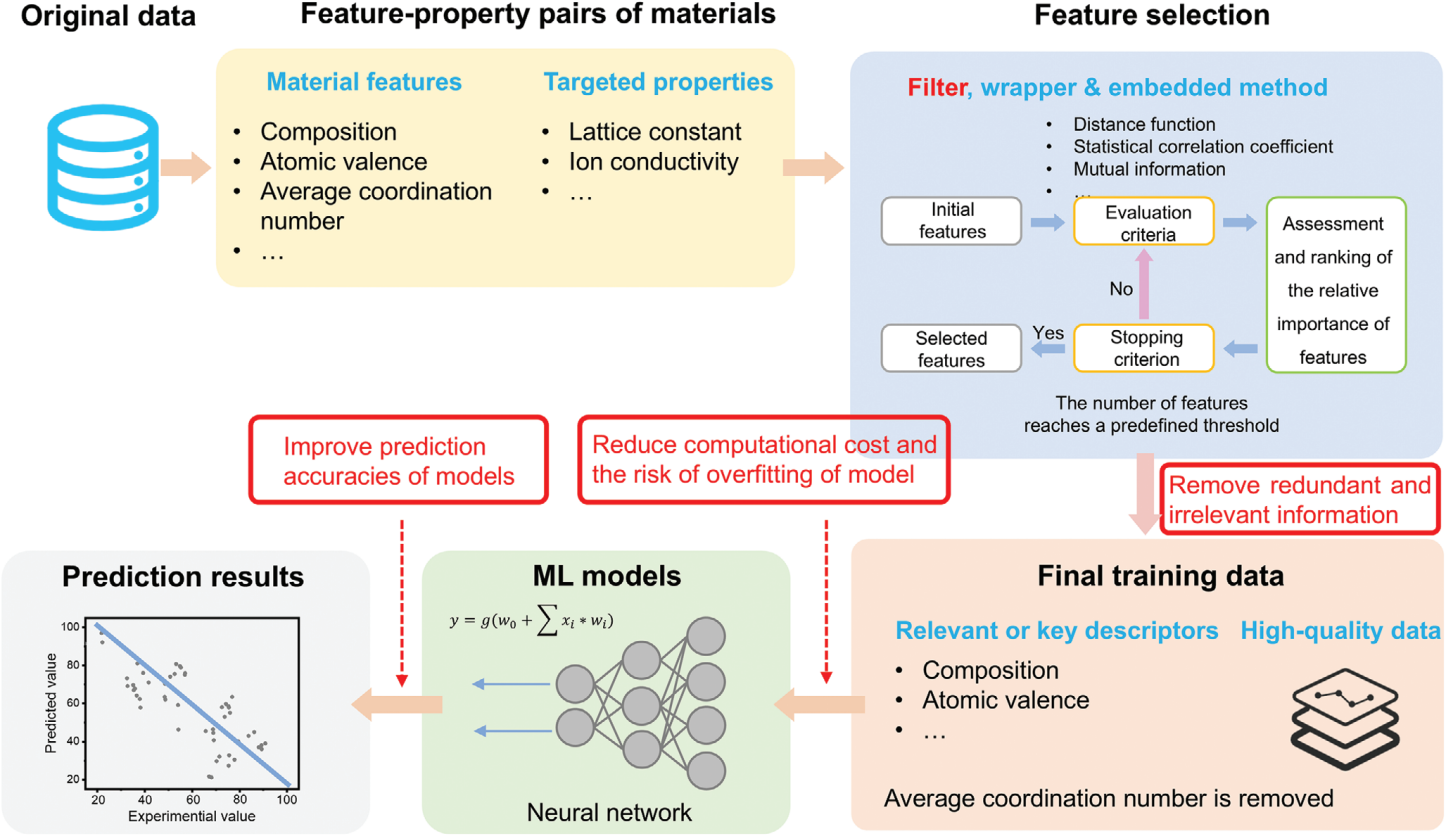
The procedure of combining ML with feature selection (FS) in predicting materials properties. FS removes redundant and irrelevant information presented in the original dataset, which reduces computational cost and the risk of overfitting for the prediction model as well as improves its prediction accuracy.

Dimensionality reduction and generation serve as the fundamental techniques of data processing, involving feature engineering and data generation that can effectively tackle the challenges originating from high‐dimensional and small sample data. Choosing the most pertinent or crucial features from the original set of features is essential for feature selection. By removing irrelevant attributes, it is able to reduce the volume of data, diminish the complexity of the model, enhance the model's generalization capability and efficiency, and minimize information loss. The commonly employed feature selection methods encompass filtering, packaging, and embedding techniques.^[^ ^53^ ^]^ The filtering method involves assessing and ranking features based on their statistical properties in order to select the top‐ranked features, including variance filtering, correlation coefficient filtering, and so on. The packaging method evaluates the significance of features based on model performance through repetitive model training, primarily including recursive feature elimination. The embedding method integrates feature selection with the model training process to automatically select the best features during model training, primarily including Lasso regression, decision tree, random forest, and other methods. Feature transformation aims to alter the distribution of features or extract new features through mathematical transformation of the original features, thereby reducing the dimensionality of existing data, improving model performance, and preserving information integrity. This can be accomplished through techniques such as principal component analysis (PCA), clustering, and linear discriminant analysis. Feature engineering contributes to enhance the accuracy and generalization performance of ML models by reducing the dimensionality of data and eliminating irrelevant features. The procedure of combining ML with feature selection in predicting materials properties is schematically shown in **Figure** **8**.

In terms of feature transformation, Wang et al. used principal component analysis (PCA) to analyze the high‐dimensional feature parameters of battery consistency, upon which they developed a multi‐parameter evaluation function and proposed a battery balancing strategy based on fuzzy control.^[^ ^61^ ^]^ Chen et al. studied incremental capacity curves using linear discriminant analysis and extracted six features from the high‐dimensional curves for each battery. They employed a shrinkage method called elastic net to select the two variables most correlated with capacity decay. Thereafter they built a battery classification model based on linear discriminant analysis, dividing the batteries into “good” and “bad” categories. They discussed the impact of prior probabilities on each battery type and the configuration that minimized the actual loss, thus improving the reliability of LIBs operation.^[^ ^62^ ^]^ In the context of feature selection, Wu et al. used a filter‐based method to select 23 key descriptors from a high‐dimensional dataset of FCC solute diffusion barriers calculated by DFT. They employed a Gaussian kernel ridge regression model to predict the diffusion barriers of all impurities on the FCC host.^[^ ^63^ ^]^ Shandiz et al. utilized an embedded feature selection method to construct 9 descriptors for 339 high‐dimensional silicate cathode material samples. They built ML models by increasing the percentage of training data, which bestows the random forests and extremely randomized trees with the highest overall accuracy, demonstrating the effectiveness and flexibility of ensemble methods in classifying crystal systems. They also used extremely randomized trees to predict the crystal systems of the aforementioned materials, which reveals that cell volume and the number of atoms in the unit cell had the most significant impact on the prediction results.^[^ ^64^ ^]^

**Figure 9:**
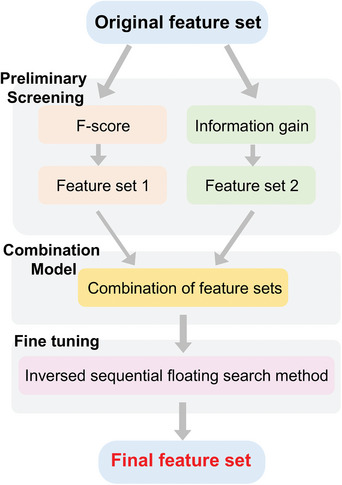
Schematic diagram of feature fusion strategy. The hybrid feature selection procedure begins with two filter models to eliminate redundant features. F‐score and information gain comprise the preliminary screening. The resulting feature sets merge into a preprocessed set for fine‐tuning, followed by a wrapper model to enhance the classification accuracy in the fine‐tuning step.

However, feature selection methods are varied and complex, and their hyperparameter optimization strategies often require manual adjustments, making it difficult for users to optimize high‐dimensional data directly. To address this issue, a feature selection fusion strategy, which is shown in **Figure** **9**, can be employed to process features from different perspectives. Hsu et al. used a computationally efficient filter to select candidate descriptors from the high‐dimension original dataset and then further optimized feature selection using a more accurate wrapper. They demonstrated that using a smaller feature set can achieve the same or better prediction accuracy, which serves as a demonstration for applying ML to high‐dimensional small‐sample data.^[^ ^65^ ^]^

**Figure 10:**
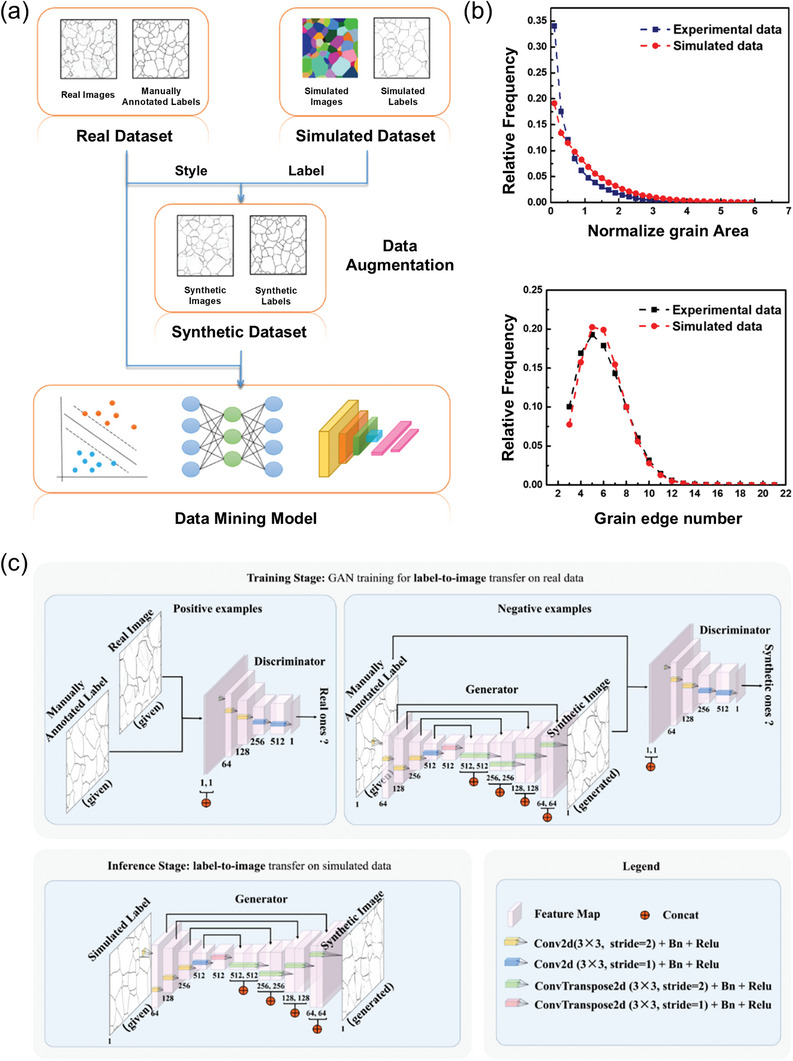
a) The strategy to create synthetic data by fusing the images obtained from simulating the physical mechanism of grain formation and the “image style” information in real images. Then the acquired synthetic data with GANs can be used as data augmentation for the training procedure of the ML model. b) The characteristic distribution comparison of experimental and simulated data. c) The image‐to‐image transfer to convert the image of a simulated label to a synthetic image, where the first row shows the training stage of the style transfer model and the second row shows the inference stage. Reproduced with permission from ref. [66] Copyright 2020 Nature.

Similar to feature selection, the method of generating new data can increase the volume of data and supply adequate data for ML, addressing the issue of high‐dimensional data from an alternative perspective. Zhang et al. utilized graph theory‐based algorithms to construct an extensive database of candidate solvent molecules. They treated the solvent molecules as graphs, where atoms are vertices and bonds are edges. Beginning with the fundamental molecules of formaldehyde and dimethyl ether, they incrementally added vertices and edges to create potential solvent molecules until reaching nine heavy atoms. This approach produced 1399 solvent molecules, demonstrating algorithm reliability through cluster analysis and experimental validation, thus facilitating further solvent research.^[^ ^51^ ^]^ Generative adversarial networks (GANs) are a confrontation framework that consists of a generator and a discriminator, of which these two elements engage in competition and enhance each other during the training phase. By reducing the discriminator's loss and maximizing the generator's loss, novel image data can be generated from a defined dataset, for the learning of more intricate distributions. GANs were employed to augment the dataset, assisting ML models in comprehending the overall patterns of intricate and multidimensional data. Song et al. combined GANs with a random forest‐based classifier to discover thousands of potential 2D materials from a large amount of heterogeneous data. DFT calculations confirmed the existence of 92 2D/layered materials, demonstrating the effectiveness of this approach in exploring the chemical design space of new materials.^[^ ^67^ ^]^ Similarly, Ma et al. used GANs to expand the original dataset of polycrystalline iron optical images, achieving comparable training results with that of models trained on real data. The evidential findings are visually represented in **Figure** **10**. Integrating ML with first‐principles calculations can produce considerable quantities of structured data, including dipole moments, highest occupied molecular orbitals (HOMOs), and lowest unoccupied molecular orbitals (LUMOs), and unstructured data such as electrostatic potential maps and charge distribution maps, which can plays a crucial role in addressing the limitations of small sample sizes in lithium battery datasets.^[^ ^68^ ^]^ This strategy mitigates the disparity between the precision and cost of MD force fields. Kim and colleagues presented a new Sparse Gaussian Process Regression (SGPR) method. This technique is particularly advantageous as it utilizes universally generated first‐principles potentials and forces. The method captures the potential and force across various distinct local chemical environments (LCEs) within a structure, factoring in diverse interatomic interactions.^[^ ^69^ ^]^ After just a few training runs, the ML potential can accurately forecast the potential energy of any provided structure. Notably, even with all training iterations, SGPR‐based ML simulations operate ≈100 times quicker than direct DFT simulations.^[^ ^70^ ^]^ Building upon this foundation, Kim et al. employed DFT alongside the ML potentials developed from the SGPR method to execute rapid simulations. They explored the mechanisms of capacity fade and voltage decay suppression triggered by aluminum doping, aiming to facilitate the creation of highly stable layered cathode materials.^[^ ^71^ ^]^ This indicates that using generated data can fully utilize the analytical and predictive capabilities of deep models without significantly increasing the amount of training data, which is particularly beneficial in fields like battery research where data is often limited.^[^ ^66^ ^]^

Data augmentation can also effectively create supplementary training samples by altering, perturbing, or merging raw data. This technique aims to enlarge the training dataset, enabling the model to comprehend various data transformations and improve its generalization capabilities. For unstructured data, particularly in image formats, prevalent data augmentation methods involving rotation, flipping, scaling, cropping, color adjustments, and noise infusion could enhance the variety within image datasets and help to mitigate the risk of overfitting. The denoising diffusion probability model (DDPM) represents a sophisticated approach in the realm of generative models, which involves a diffusion process driven by iterative denoising. In essence, it reverses the progression by moving the sample from the initial image distribution to a Gaussian distribution, thereby establishing a model. This reflects the process of observing image data gradually contaminated by noise and subsequently learn to eliminate the noise, ultimately restoring the image data to its original state and generating a fresh appearance. This methodology empowers the transition from the complete Gaussian distribution to the image distribution, facilitating the generation of high‐quality samples. Moreover, the model training demonstrates remarkable stability, suggesting its promising application in the domain of battery image processing. In addition, it is crucial to analyze time series data for predicting battery capacity degradation and cycle life within the battery domain. In addition, utilizing a diffusion model for time series data processing can address challenges like data scarcity from diverse sources, improving prediction stability and interpretability.^[^ ^72^ ^]^

### 4.4 Modeling and Evaluation—A Solution for the Data Issue of Small‐Sample Size

The modeling and evaluation are fundamental means in ML for the problem of small‐sample size data. The primary objective is to determine the model that most effectively represents the data within the model space through iterative training and evaluation. Common models consist of individual models and composite models. In the past, ML in the battery field primarily relied on training, verifying, and predicting data using single models, which is due to the fact that the single model demonstrates strong applicability in addressing battery data under specific conditions, while it unavoidably presents certain limitations.^[^ ^73^ ^]^ Duquesnoy et al. trained and tested an ML classifier, namely the Gaussian naive Bayes algorithm, with a small experimental dataset to predict whether the electrode was homogeneous or heterogeneous based on manufacturing parameters. This provides a valuable tool for optimizing the next generation battery electrodes, yet this model is sensitive to the expression of input data.^[^ ^74^ ^]^ Zhao et al. introduced a prediction framework called *E*_a_‐In‐SSS ML based on a hierarchical encoding crystal structure (HECS) descriptor. They established prediction models for E_a_ (activation energy) using partial least squares (PLS) analysis, achieving determination coefficients (*R* ^2^) of 0.887 and 0.02 eV for the training dataset, and 0.820 and 0.02 eV for the testing dataset, respectively. This confirmed the predictive ability of the HECS descriptor and its good potential in designing advanced solid electrolytes. Even though the single model demonstrated some feasibility with small‐sample data, it is not suitable for high‐dimensional data.^[^ ^75^ ^]^

**Figure 11:**
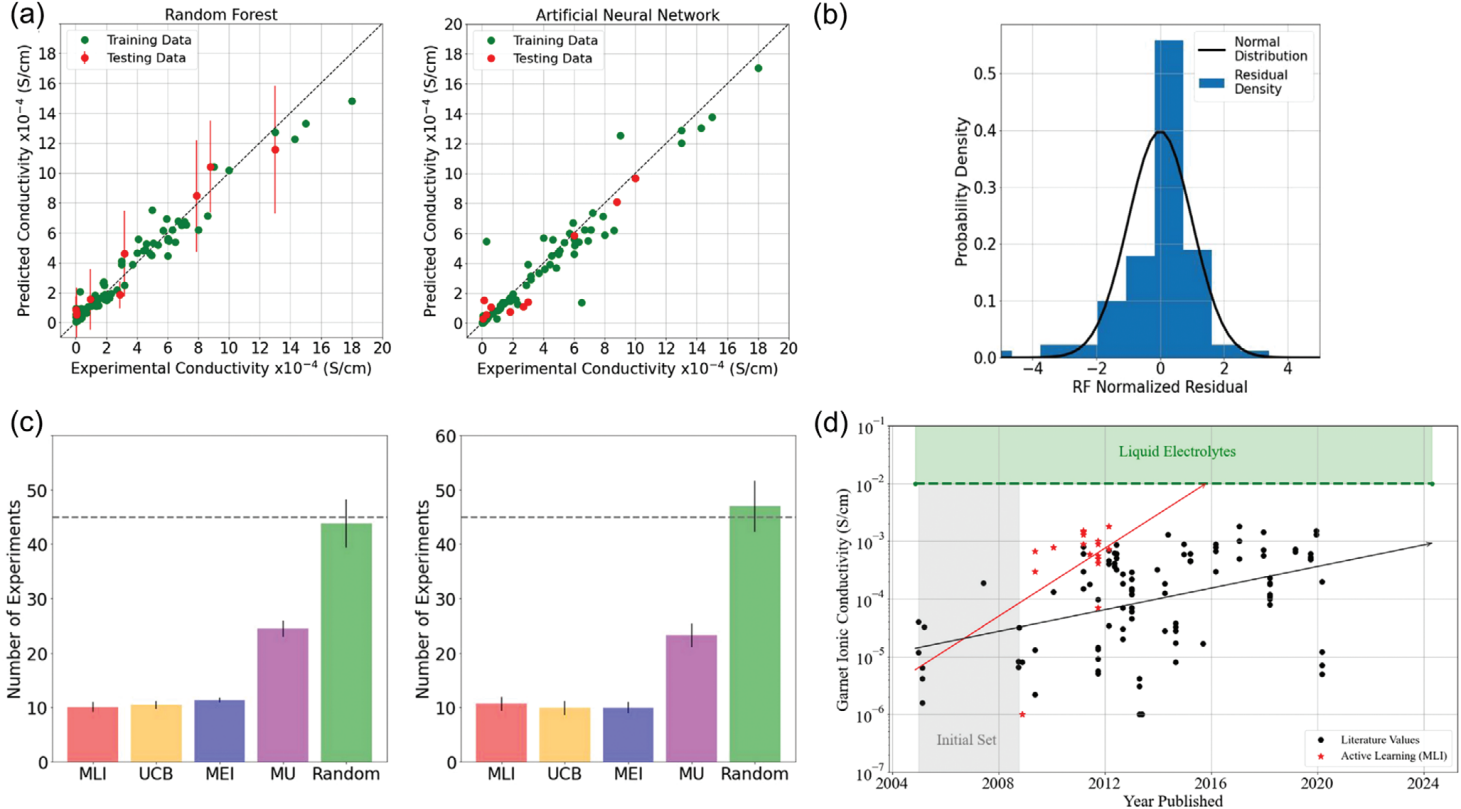
a) Parity plots for prediction of Li‐ion conductivity in garnets using a random forest with uncertainty and b artificial neural network. Uncertainty estimates are included for the random forest. b) Probability densities of normalized residuals computed via tenfold cross‐validation for the random forest. c) Mean number of predictions per information acquisition functions for the 30‐trial runs of the active learning model. The dashed line represents half of the experiments. In the model, samples with 10 experiments at random and 10 oldest experiments were included, respectively. d) A possible timeline of published experiments informed with the maximum likelihood of improvement acquisition function. Each point represents one material and when it was published. Black points represent when literature values were published. Red points indicate points explored by the function before finding the optimal candidate. Reproduced from ref. [76] Copyright 2020 IOP science.

In the battery field, the data is often small, along with multi‐sources, heterogeneous, or high‐dimensionality, of which these characteristics are interconnected. The solution to such data with high complexity and diversity cannot be achieved through a singular model. The limitations of individual models in handling lithium battery material data necessitate the emergence of a model fusion strategy to expand their applicability ranges. By combining the merits of multiple individual models, a better generalization ability can be achieved, leading to superior performance in data processing tasks. Model fusion is a way of integrating sub‐models according to certain methods or frameworks, with different classifications based on the dataset, algorithm, and fusion strategy. For instance, the fusion method can be classified as homogeneous or heterogeneous based on whether the sub‐models are constructed using the same algorithm. Homogeneous fusion, which is based on the same algorithm, holds promising prospects in handling lithium battery material data. Verduzco et al. have demonstrated that an active learning approach, based on random forest prediction models, can be used for experimental design with small‐sample and imbalanced experimental data of Li‐ion conductivity in lithium lanthanum zirconium oxide (LLZO) garnets (**Figure** **11**).^[^ ^76^ ^]^ Wu et al. used an extreme gradient boosting algorithm to identify features for building a battery degradation model, which proves that this method has high estimation accuracy and generalization ability, with a small error of about 2% for batteries in actual electric vehicles.^[^ ^77^ ^]^ In heterogeneous fusion, combining different algorithms provides greater flexibility without a fixed framework, which allows for broader prospects in data processing, especially in the field of battery research. Nagulapati et al. conducted a study on battery capacity and health estimation using Gaussian process regression (GPR) and support vector machine (SVM) models, which significantly enhanced the predictive accuracy of single and multiple sensor data by combining multiple battery datasets.^[^ ^31^ ^]^ Similarity, Tian et al. developed a deep neural network (FNN) SOC estimation method based on convolutional neural network (CNN) and recurrent neural network (RNN) with a model fusion strategy. By training the model with three groups of batteries and thereafter testing it with the fourth group, they achieved accurate SOC estimation for small‐sample data charging processes.^[^ ^49^ ^]^

**Figure 12:**
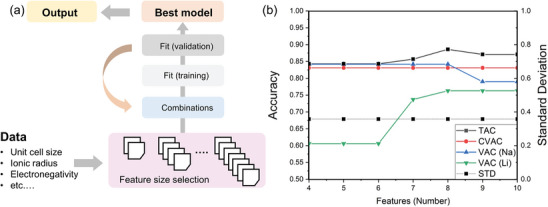
a) Model evaluation process used in our study. Combinations of different‐sized feature sets are systematically evaluated for training and validation accuracies, with the best‐performing combination at each feature size recorded. b) Results from systematic feature evaluation, displaying the best model performance across permutated feature sets of size n, where *n* is between 4 and 10, inclusively. Reproduced from ref. [78] Copyright 2020 IOP science.

Model evaluation methods primarily encompass accuracy, precision, recall, F1 score, area under curve, Log Loss, and the generalization error assessment. This evaluation is done by assessing the model's ability to work with different structured and unstructured data disadvantages, which involves the application of datasets that are typically divided into training, testing, and validation sets. The training set is used to train the model by fitting a linear regression model to the data samples. The testing set is then used to evaluate the final model's performance. The validation set, which is a separate portion of the training set, is used to adjust the model's hyperparameters and make an initial assessment of its capabilities. Iterating the model in this manner can optimize its parameters, improve its reliability, and enhance its generalization ability for data issues. Cross‐validation serves as a vital technique for model evaluation and selection in ML. It assesses model performance by partitioning the dataset into several subsets. This approach helps to mitigate overfitting and enhances the model's generalization capabilities. To counteract randomness in training‐test segmentation scores, it is prudent to repeat the dataset partitioning process multiple times. By averaging the test set results, one can ensure a comprehensive representation of target variables, thereby allowing for effective performance evaluation. K‐Fold Cross Validation generates a test set that uniformly encompasses the entirety of the target variable range. Initially, the dataset divides randomly into *k* equal and mutually exclusive subsets. The model undergoes training on *k*‐1 subsets while testing on the remaining one, a process that repeats k times, each time selecting a different subset as the test set. Consequently, this method produces *k* sets of training/testing data, from which training and testing iterations occur, with the final evaluation reflecting the average of the *k* test outcomes.^[^ ^79^ ^]^ In addition, continuously developing models and assessment methods can concurrently address multiple data problems. Kedar et al. designed a logistic regression‐based model for a target compound with a limited dataset of 70 NASICON‐examples, realizing a validation accuracy of over 84% (**Figure** **12**). And through a systematic permutation‐based features evaluation process, they reduced the number of considered features from 47 to 4,5,6,7,8,9,10 respectively. Through an analysis of training accuracy (TAC), cross‐validation accuracy (CVAC), validation misclassification rate (VAC), and model standard deviation (STD) across various feature sets, it was observed that a feature size of 8 attained the highest training accuracy of 0.886, corresponding to a reduction of over 83%, which simultaneously improves the model performance.^[^ ^78^ ^]^ Sendek et al. demonstrated that the generalization error might decrease as the model captured more details of the distribution. When working with small‐sample data, the effective measure is to find a model that achieves a balance between under‐fitting and over‐fitting. Under‐fitting occurs when the model fails to capture enough variance or dimensionality of the data, which can be mitigated by increasing the flexibility or complexity of the model to reduce its generalization error.^[^ ^80^ ^]^ On the other hand, over‐fitting happens when the model is too flexible or complex for the underlying structure of the training data. In this case, reducing the model's flexibility or complexity can improve its ability to generalize new data. By employing this approach, we can assess the model's accuracy in small‐sample data through the measurement of generalization error. Simultaneously, the assessment outcomes of the model can be utilized to constantly fine‐tune the model's parameters, resulting in a model with robust generalization capabilities. This process also enables the exploration of potential information and relationships within limited sample data, which effectively tackles the underfitting and overfitting challenges associated with small sample sizes, enhancing the effective utilization and extraction of restricted data.

**Figure 13:**
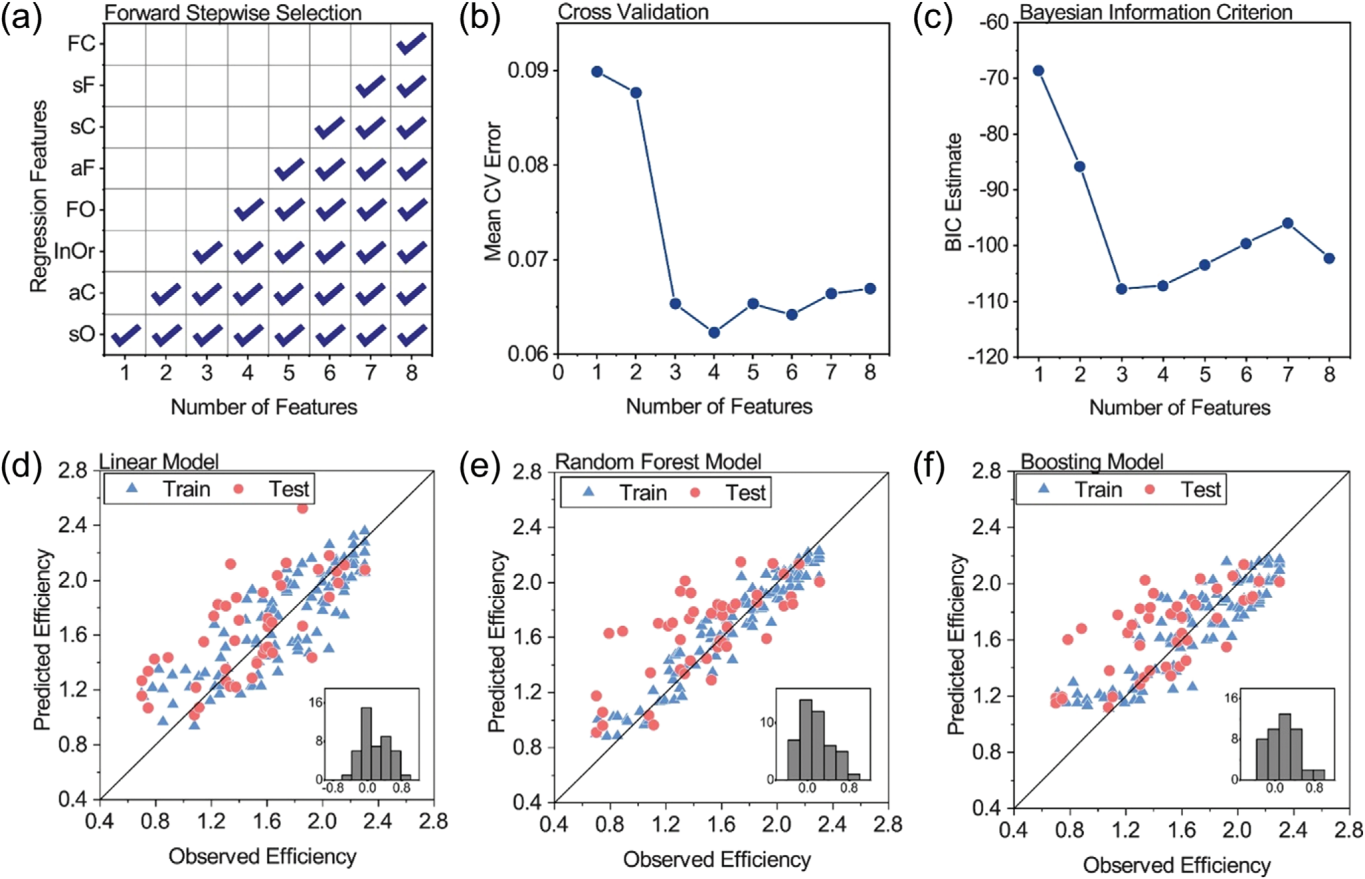
Model development. a) Reduction of feature space using forward stepwise selection. b and c) Cross‐validation errors and BIC estimates reveal that the four‐feature model shows the smallest errors. d–f) Plots of predicted efficiency versus observed efficiency for the four‐feature linear model, random forest model, and boosting model, respectively. Insets show histograms of residuals (predicted–observed) for the test data. Reproduced from ref. [81] Copyright 2023 PNAS.

As mentioned above, evaluating the extracted descriptors and filtered features is another crucial step in the ML process. Assessing the correlation between descriptors and target variables can help identify more reliable descriptors, which is of vital importance for model interpretation. Xie et al. conducted sensitivity analysis to determine the importance of each descriptor in model construction, gaining insights into the “black box” of generating ANN models. By selecting the top two descriptors, they accurately predicted four types of cathode materials from 6871 data points, demonstrating its exceptional performance.^[^ ^82^ ^]^ Cui et al. applied a forward stepwise selection technique to assess feature significance based on the small‐sample data, revealing that sO stands out as the most crucial factor in elucidating the logarithmic Coulombic efficiency (LCE). The process involved iteratively enhancing feature combinations based on the residual sum of squares in a regression model. Starting with a single feature (sO) and progressively adding others, the algorithm advanced from a model centered on sO to another one incorporated with seven additional features. The analysis underscores the pivotal role of solvent oxygen content in explaining LCE, which suggests that reducing sO could enhance the Coulombic efficiency, leading to noteworthy advancements in lithium metal battery research (**Figure** **13**).^[^ ^81^ ^]^

### 4.5 Domain Knowledge Embedding Data Processing—An Effective Supplementary Strategy for Resolving Data‐Centric Challenges

**Figure 14:**
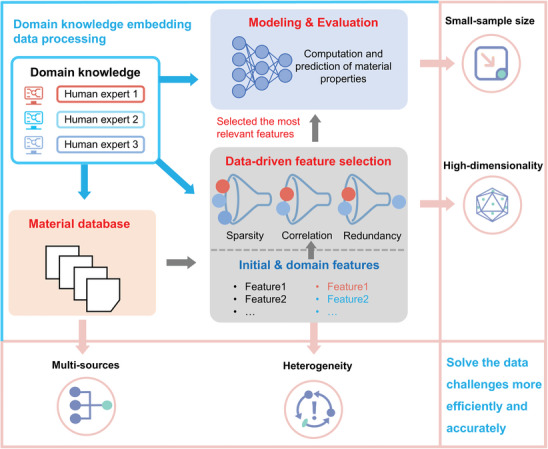
The schematic diagram of integrating domain knowledge into the ML process to solve the main data challenges more efficiently and accurately.

The interpretability of ML outcomes in the battery industry is impacted by a degree of randomness. This randomness arises from uncertain data quality and the subjective domain knowledge held by researchers with various backgrounds.^[^ ^36a^ ^]^ Most models heavily rely on sample data and lack guidance from domain knowledge, which is one of the main reasons that causes the non‐accuracy of ML with data‐driven. Addressing data quality concerns is crucial, but it's also essential to understand how domain expertise influences data processing and ML. Integrating domain knowledge into the ML process could enable the aforementioned data processing methods to address the four primary data challenges more quickly, efficiently, and accurately (**Figure** **14**).

The problem of data inconsistency can be solved by introducing integrity to constrain the attribute values of related entities. For the confliction of multi‐sources data, the real value of each entity can be determined by trusting reliable conflict sources, indicating the important significance of domain knowledge in data processing.^[^ ^83^ ^]^ The different types of descriptors are designed to adapt to specific data and algorithms, thereby improving the efficiency of ML models and effectively addressing the diverse sources of material data. Even though there are existing integrated descriptor calculation tools, it is necessary to propose a general descriptor extraction scheme with domain knowledge that can be applied to any targeted property, due to the diversity and complexity of factors affecting lithium battery material properties. This will facilitate the efficient and accurate data input throughout the entire process of ML to solve related problems. Ward et al. have made promising progress in this direction by proposing a universal descriptor framework based on the physical and chemical properties of materials, which allows them to establish accurate models for different properties, such as the electronic properties of crystalline compounds and the glass formability of metallic alloys. This framework has been successfully applied in the prediction of material properties in electrochemical energy storage.^[^ ^84^ ^]^ Furthermore, Wei et al. are also of the opinion that incorporating domain‐specific knowledge into ML can effectively address the challenge of data from multi‐sources, which then substantially increases the prediction accuracy of the model.^[^ ^85^ ^]^ Liu et al. presented a technique for selecting high‐quality descriptors by incorporating domain‐specific knowledge. This technique transforms the domain knowledge of highly correlated features into non‐co‐occurrence rules (NCORs), quantifying the degree to which feature subsets violate these rules. Thereafter, they designed an optimization process for selecting descriptors by leveraging swarm intelligence algorithms. Experimental results of seven datasets demonstrated that this approach could enhance the prediction accuracy and interpretability of ML models.^[^ ^86^ ^]^

While the above studies have shown that managing data quality can address data heterogeneity and improve the accuracy of ML models, the current data processing methods mostly focus on detecting heterogeneity from a single perspective. Given the complexity and multiple systems involved in lithium battery material design, it is challenging to comprehensively evaluate and manage data heterogeneity. Additionally, material data is highly specialized, while the data preprocessing methods rely solely on statistical analysis, which overlooks the importance of domain knowledge in improving data quality. Therefore, it is necessary to establish a general data quality governance framework specialized for ML applications in the field of battery materials. This framework should integrate domain knowledge to effectively manage heterogeneous material data, enabling real‐time detection and comprehensive control of data quality throughout the entire process. This will ultimately lead to more accurate data analysis and reliable decision‐making.

Besides, it is important to note that incorporating domain knowledge into feature selection and transformation methods as well as selecting or constructing descriptors with physical meanings, can help reduce data dimensionality as well as build simple and accurate ML models. Liu et al. introduced a technique known as Data‐driven Multi‐layer Feature Selection (DML‐FS), which sought to create a prior model of feature importance using domain expert knowledge applied to initial features in the material database. This model was then used to guide the process of selecting relevant features. By using three hierarchical processing layers, the challenges of data heterogeneity, irrelevance, and dimensionality were effectively managed. This approach ensured that the selected features were both highly discriminative and strongly correlated with the targeted attribute.^[^ ^87^ ^]^

**Figure 15:**
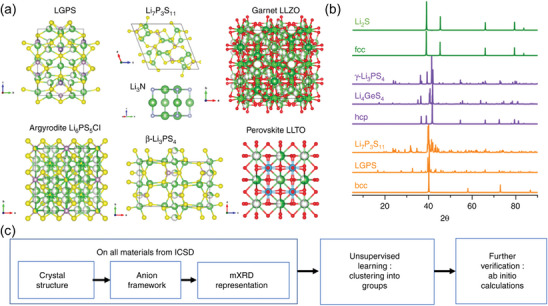
Schematic illustration of the unsupervised discovery of solid‐state Li‐ion conductors. a) Crystal structures of known solid‐state Li‐ion conductors (SSLCs), showing a large diversity of structure and chemistry. b) mXRD patterns of selected materials in comparison to those of ideal fcc (face‐centered cubic), hcp (hexagonal close‐packed), bcc (body‐centered cubic) lattices. c) Workflow of an unsupervised learning guided discovery of SSLCs. Reprinted with permission from ref. [88] Copyright 2019 Nature.

Moreover, Sendek et al. emphasized the significance of using physics‐guided ML methods to enable success based on small data, rather than relying on arbitrary requirements for dataset size.^[^ ^80^ ^]^ Lee et al. constructed a neural network regression model that considered both electronic properties and molecular structural parameters. This model successfully established a relationship between the chemical stability of over 90 available electrolyte solvents and representative redox mediators, providing valuable guidance for the development of stable electrolyte/redox mediator pairs for lithium‐oxygen batteries.^[^ ^89^ ^]^ Sendek et al. combined high‐throughput first‐principles calculations with a logistic regression model, which enable them to identify 20 ion conductivity descriptors associated with crystal local atomic arrangement and chemical environment features from a training set of 40 Li‐containing compound structures with experimental ionic conductivity. Using these descriptors, they screened 21 potential solid‐state electrolyte materials from a pool of 12381 Li‐containing compounds.^[^ ^90^ ^]^ Zhang et al. employed clustering methods in ML to identify 16 solid‐state electrolytes with predicted conductivity ranging from 10^−4^ to 10^−1^ S cm^−1^ using ab initio molecular dynamics simulations (**Figure** **15**). This demonstrated the feasibility of ML to discover materials within a vast materials space, even with limited property data.^[^ ^88^ ^]^ These studies clearly demonstrate the importance of incorporating domain knowledge into the ML process, particularly in data processing and model construction. Specifically, embedding domain knowledge in the materials domain should not only focus on target definition, data preparation, and data‐driven algorithms validation but also should look into the entire ML workflow. We anticipate that the idea of optimizing algorithms through the integration of domain knowledge into the proposed model fusion strategy, including ML models, strategies, and algorithms, has the potential to develop a universal and automatic method to select ML algorithms for various materials tasks and characteristics.

### 4.6 Comparisons of Data Processing Methodologies

In the domain of ML, it is essential to evaluate various algorithms and strategies for data processing. By assessing metrics such as accuracy, precision, recall, and F1 score, one can determine the optimal model. This comparative analysis reveals which features most significantly affect model predictions, thereby highlighting the importance of data structure and characteristics. Furthermore, by analyzing model performance on training and validation datasets, one can detect the likelihood of overfitting. Some models may excel with training data but falter with new data. This analysis uncovers the promise of ensemble learning, which enhances overall prediction accuracy by combining multiple models' outputs. In comparing several models, the identification and removal of underperforming options becomes more efficient, expediting the development process and curtailing resource expenditure. Additionally, this comparative approach provides insights into model resilience amidst shifts in data distribution or in noisy environments.

It is crucial to emphasize that determining the prerequisites for assessing the pros and cons of different strategies requires the selection of a consistent dataset. Yet, directly comparing data processing techniques across differing datasets remains challenging. Thus, it is crucial to select the most appropriate data processing method or to synergize these methods based on specific task requirements. For instance, when predicting the healthy lifespan of data and comparing data processing techniques: In dataset 1, the Root Mean Square Error (*R*_MSE_) of the Convolutional Neural Network (CNN) extraction method is 0.0193, with an R‐squared (*R* ^2^) value of 0.9913. The *R*_MSE_ and *R* ^2^ values of the Attention CNN‐LSTM method, which integrates domain knowledge and model fusion, are 0.0149 and 0.9949, respectively. In contrast, the CNN‐LSTM method that employs only model fusion exhibits *R*_MSE_ and *R* ^2^ values of 0.0221 and 0.9646, respectively.Moving to dataset 3, the CNN‐trained model (extraction method) shows an *R*_MSE_ of 0.0151 and an *R* ^2^ of 0.9947. Once again, the prediction results from the Attention CNN‐LSTM method yield *R*_MSE_ and *R* ^2^ scores of 0.0149 and 0.9949, respectively. The CNN‐LSTM model, utilizing solely model fusion, records *R*_MSE_ and *R* ^2^ values of 0.0232 and 0.9875. These results clearly demonstrated that integrating both domain knowledge and model fusion could significantly enhance the consistency of the prediction with less error, compared to that solely employing model fusion.^[^ ^91^ ^]^

It is worth noting that the comparison of data processing strategies between different datasets is meaningless. The Backpropagation Neural Network (BPNN) represents a multilayer feedforward network model. In this model, input signals flow directly, lacking feedback mechanisms. Wu and colleagues applied the Firefly Algorithm to BPNN for predicting the SOC of lithium iron phosphate batteries. They used voltage and discharge current as input variables, resulting in a prediction error of 1.54.^[^ ^92^ ^]^ Following this, a Radial Basis Function (RBF) model was developed, which includes strategies for model evaluation. Notably, the K‐means clustering algorithm determined the number of hidden layers in the RBF network. This approach optimized the architecture, reducing the prediction error to around 0.5%.^[^ ^93^ ^]^ The integrated model utilizing Long Short‐Term Memory Networks (LSTM) and Recurrent Neural Networks (RNN) adeptly estimates the SOC of batteries under diverse environmental temperatures. When temperatures increase from 10 to 25 °C, the model attains a Mean Absolute Error (MAE) of 1.606%.^[^ ^94^ ^]^ To address complex operational conditions, the integration of Autoencoder Neural Networks with LSTM networks significantly enhances SOC estimation accuracy. The Autoencoder Neural Network extracts essential features from lithium‐ion batteries, while the LSTM network delivers precise predictions. This method's effectiveness under Dynamic Stress Test (DST) and Fuel Cell Uncertainty Scenario (FUDS) conditions has been validated, resulting in an MAE below 0.94%.^[^ ^95^ ^]^ Herle et al. highlight that the scarcity of datasets hampers the progress in SOC estimation. They suggest a model fusion strategy that combines two neural networks, facilitating reliable estimates even with limited data. When comparing different data scenarios, the Mean Absolute Error (MAE) of the Feedforward Neural Network (FNN) feature extraction approach remains below 2.23% under DST and FUDS conditions.^[^ ^96^ ^]^ In contrast, the model fusion strategy incorporating a Convolutional Neural Network‐Gated Recurrent Unit (CNN‐GRU) achieves an MAE lower than 1.95%. This indicates that various data processing techniques do not automatically rank in terms of superiority. Instead, their effectiveness depends on choosing the most suitable method for each distinct dataset.^[^ ^97^ ^]^ The previous analysis reveals that extraction and model fusion strategies produce different degrees of accuracy across various datasets. It is essential to highlight the significance of comparing distinct data processing methods. Nonetheless, fundamentally, no single strategy is inherently superior or inferior based on the mathematical nature of data and its manipulation. A method that successfully tackles data issues is deemed valid, whether it employs a singular approach or multiple strategies in parallel.

## 5 Conclusions and Perspectives

### 5.1 Conclusions

When employing ML techniques for data analysis, the ultimate level of accuracy in prediction is generally defined by the quality of the data. The algorithms, strategies, and models are usually served as means to estimate and approach the upper limit of prediction, which is like a relation between essence and phenomenon. In this paper, we systematically discuss the corresponding strategies to address these challenges of lithium battery materials data, which include classification and extraction, screening and exploration, dimensionality reduction and generation, modeling and evaluation, and domain knowledge embedding data processing.

1. Classification and extraction: Classifying lithium battery materials data from various sources and extracting data relevant to targeted attributes, particularly descriptors, constitutes an effective approach to address the challenge of multi‐source data. Multi‐source data will naturally result in multi‐source models. Classifying the models and selecting appropriate models for integration not only enhances the model's performance but also lowers the training expenses, which signifies the future direction of development. Employing classification algorithms to scrutinize the data aids in enhancing comprehension of the internal composition and arrangement within the problem domain, which unveils the concealed patterns and associations in the data as well as offers direction for feature engineering and descriptor extraction, thus facilitating the lithium battery materials design.
2. Screening and exploration: Screening and exploration represent fundamental technologies in lithium battery materials data structure management. They primarily concentrate on filtering and processing anomalous data, selecting a standardized data format beneficial for ML, organizing data structures, and investigating potential correlations. These methods promote both the credibility and interpretability of the data, and subsequently enhance its reliability. Ultimately, high‐quality data can significantly enhance the practical applicability of ML models and facilitate the exploration of the relationship between material structure and performance. Besides the explicit linkages within data, it is crucial to delve into its implicit associations. Exploring the potential connections within data and selecting crucial descriptors or features stands as a vital method for addressing data challenges, representing a promising avenue in the realm of battery research.
3. Dimensionality reduction and generation: Dimensionality reduction and generation serve as the fundamental techniques for lithium battery material data processing, involving feature engineering and data generation to effectively tackle the challenges arising by high‐dimensional and small sample data. Feature selection entails choosing the most pertinent or crucial features from the original set of features. By removing irrelevant attributes, it is able to reduce the volume of data, diminish the complexity of the model, enhance the model's generalization capability and efficiency, minimize information loss, as well as improve the accuracy and generalization performance of ML models. It is of significant importance to mention that transforming small lithium battery material data into more features and reducing dimensionality through data abstraction may improve model interpretability and reliability. Generating novel structured and unstructured data can offer substantial assistance for ML as well as address the issue of limited data sample size from a different angle.
4. Modeling and evaluation: The processes of modeling and evaluation are fundamental means in ML. The primary objective is to identify the model that most effectively represents the data within the model space through iterative training and evaluation. Common models consist of individual models and composite models, among which the solution to data complexity and diversity cannot be achieved through a singular model. By combining multiple individual models, a better generalization ability can be achieved, leading to superior performance in data processing tasks. Homogeneous fusion, which is based on the same algorithm, holds promising prospects in handling lithium battery material data. On the other hand, developing fusion strategies across heterogeneous models can effectively utilize lithium battery material data and algorithms that are not compatible with the homogeneous one, resulting in enhanced applicability and interpretability of the fusion model. In addition, the assessment outcomes of the model can be utilized to constantly fine‐tune the model's parameters, resulting in a model with robust generalization capabilities. Evaluating the extracted descriptors and filtered features is also a crucial step in the ML process. Assessing the correlation between descriptors and targeted variables can help to identify more reliable descriptors, which is of vital importance for model interpretation.
5. Domain knowledge embedding data processing: The interpretability of ML outcomes in the battery industry is heavily impacted by a degree of randomness. This randomness arises from the uncertain lithium battery material data quality and subjective domain knowledge held by researchers with various backgrounds. Addressing data quality concerns is crucial, nevertheless, it's also essential to understand how domain expertise influences data processing and ML. Converting domain expertise into measurable descriptors that hold physical significance, such as establishing connections with other physical parameters, defining the conditions to reproduce comparable data, extracting theoretical or empirical standards from the domain‐specific knowledge as well as incorporating them into the objective function, constraint conditions, or structural symbol database of ML algorithms, can effectively address the data issues, which offers valuable insights for battery design.

### 5.2 Perspectives

**Figure 16:**
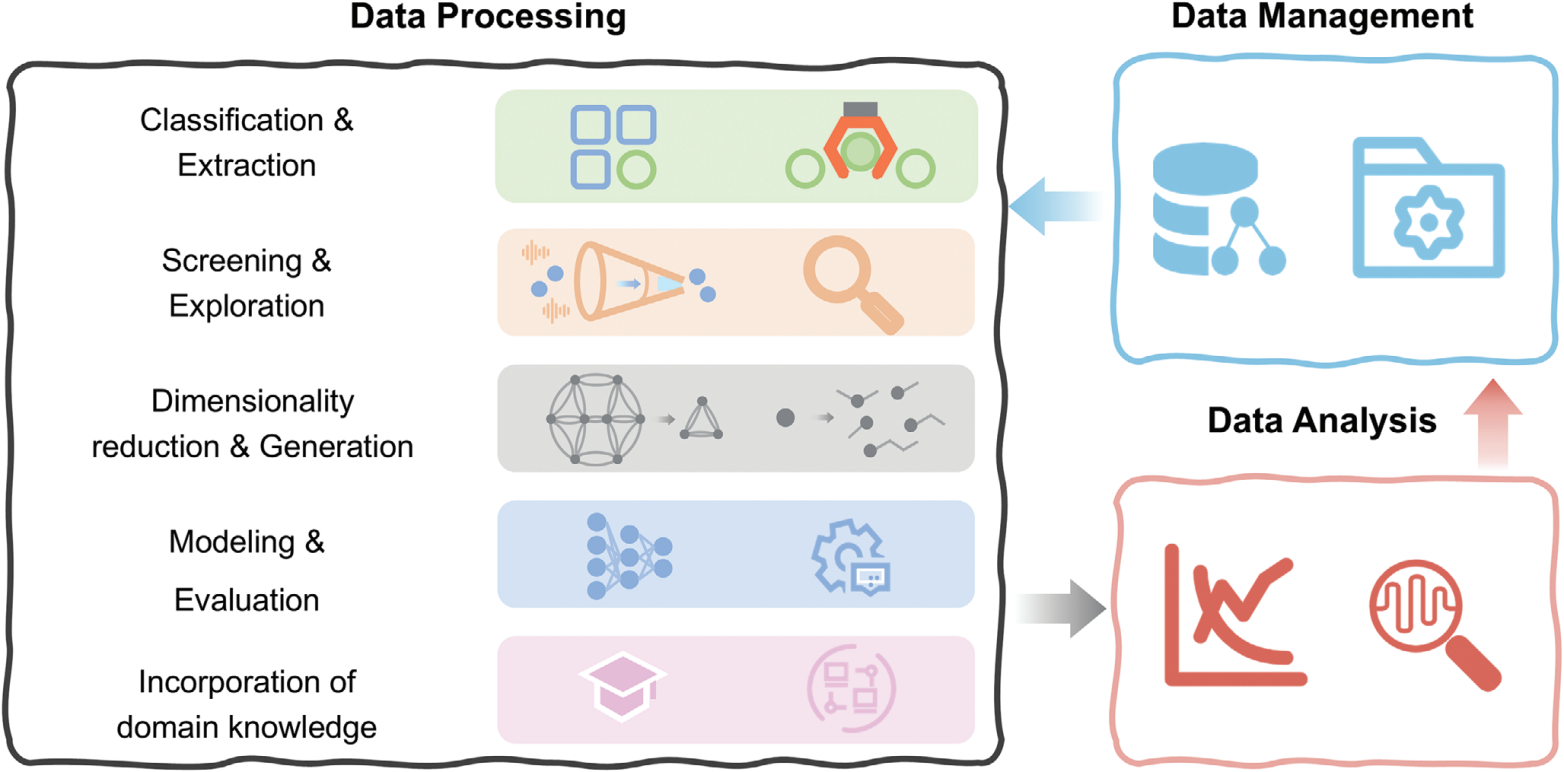
The schematic illustration of the data engineering process with data management, data processing, and data analysis strategies.

By using the data processing strategies described above, data quality, model reliability, and interpretability can be enhanced, which provides crucial guidance for lithium battery materials design. In addition to the data processing strategy, other possible strategies for addressing data quality, including data management and data analysis methodologies, also need to be emphasized, for which these three strategies constitute a comprehensive and sustainable data engineering process. Initially, combine and standardize datasets from multiple sources through data management methods. Subsequently, implement suitable strategies during the data processing stage to improve data quality, where the high‐quality data was refined for training ML models. Lastly, apply targeted techniques to analyze and extract valuable information from the predicted results of ML. The information gained can further augment the original dataset (**Figure** **16**).

For data management techniques, the tasks of classifying, filtering, and managing the data as well as establishing a unified database or standardized protocols are essential for improving efficiency in data processing and enhancing the quality of candidate data through domain knowledge. These databases are generated through various calculations and experimental databases using methods such as DFT calculations, MD simulations, and PFM simulations, which greatly promote the application of ML in research fields such as material performance prediction modeling, material screening, and new material development. It is also crucial to integrate and share data from different sources based on the FAIR principles to improve its availability. Additionally, it is of urgent requirement to develop high‐quality detection methods for electrochemical energy storage material data as well as establish unified requirements for data transmission. Validating data reliability or modifying the data empirically during transmission based on known mechanisms is expected to effectively address the challenge of connecting data between multiple‐scale models.

For data analysis methodologies, it plays an essential role in advancing our understanding of various concepts within the battery domain, such as battery reaction kinetics, space charge layers, coordination chemistry, and phase field simulations. It has the potential to be utilized for examining the intricate relationship between the micro structure‐macro performance of batteries. This includes the investigation of various processes, such as the mode inversion at the solid‐liquid interface, the chemistry of the bulk and surface, the kinetics of the reactions, the local chemistry, the dendrite growth problem, and the nonlinear kinetics at the interface. The utilization of data analysis not only assists in experimental design and computational techniques optimization to investigate various intricate relationships, but also enhances the theoretical knowledge, analysis, and testing technology in the field. Specifically, it can promote the advancement of methods for analyzing electrochemical impedance spectroscopy, x‐ray photoelectron spectroscopy, Raman spectroscopy, scanning transmission x‐ray microscopy, distribution of relaxation time, battery capacity degradation, and lifespan prediction, which greatly promotes the lithium battery material design and associated technologies and methodologies.

Besides, the data process strategies also hold significant application value across numerous research directions and fields. For example, classifying battery data using domain knowledge also enhances the efficiency of managing the production and recycling of batteries holistically, offering promising applications in the field of waste battery recycling technology. In addition, the extraction of multidimensional descriptors from structured data of electrode materials, or unstructured data obtained through methods such as TEM and phase‐field simulation may be the future direction of battery design. The integration of ML with first‐principles calculations, such as DFT, can also produce considerable quantities of structured data, including dipole moments, highest occupied molecular orbitals, and lowest occupied molecular orbitals, and unstructured data such as electrostatic potential maps and charge distribution maps which can play a crucial role in addressing the limitations of small sample sizes in lithium battery datasets. Developing new data screening methods, exploring chemical structures and properties, as well as utilizing mathematical statistics, topology, and other disciplines in ML, could accelerate the development of new ML models. It is notable that applying a diffusion model for the analysis and prediction of time series data is anticipated to have a significant impact on forecasting battery capacity decay and cycle life. The time series data generated by the diffusion model also shows significant promise in forecasting battery lifespan. Moreover, the analyzed image data plays a critical role in elucidating the evolution of battery theory models, which offers instructive insights into the field of battery technology. And developing new data screening methods, algorithms, and standards for assessing data quality aims to create a unified data analysis framework for lithium battery material data, of which the framework will also contribute to identify reliable optimization strategies and model parameters. It is notable that domain knowledge is crucial for data‐driven models and holds promising potential for model‐driven strategies such as applying ML to address partial differential equations and Schrödinger equations. In general, addressing these data challenges via the aforementioned data process strategies will significantly advance lithium battery material development, swiftly predict and efficiently create new materials to meet demand, support material science and technology, and foster intelligent development in material research and production applications.

In fact, data processing can optimize both the front data management and back data analysis. Developing back‐end data applications will further advance the integration of front‐end data management techniques, and thereby enhance comprehension of data processing methods, which establishes a virtuous cycle of the data engineering process. Most importantly, one of the core issues in ML is to address data quality issues. In theory, with a sufficiently large sample size, these issues can be effectively resolved, which leads to the following two main directions in ML development for data processing:

1. Most current data generation methods utilize graph neural networks for unstructured data generation like images. Feature extraction and analysis are then conducted on these images, with the aim to achieve desired outcomes. However, efficient methods for generating structured data are absent, highlighting the critical necessity for techniques capable of producing substantial amounts of structured data. This represents a promising pathway for advancing ML methodologies in the battery field. Leveraging domain expertise and artificial intelligence could significantly promote the advancement of these techniques.
2. Essentially, the current electrochemical data originates from both experimental and computational sources based on various theories and models. Subsequently, ML is leveraged in a data‐centric approach to analyze the potential relationships between structure and performance, where a substantial volume of data is needed. In contrast to data‐focused methods, the model‐driven strategy allows for adjustments to the initial conditions within known models, which facilitates the acquisition of a significant amount of dependable data, and potentially leads to the establishment of a cohesive physical and chemical model. Moreover, these models could dramatically advance the progression of ML techniques and the field of electrochemistry.

## Conflict of Interest

The authors declare no conflict interest.
